# *SMN1* variants identified by false-positive SMA newborn screening tests: Therapeutic hurdles and functional and epidemiological solutions

**DOI:** 10.1016/j.ajhg.2026.01.012

**Published:** 2026-02-12

**Authors:** Brunhilde Wirth, Joyosmita Das, Heike Kölbel, Shuxiang Goh, Michelle A. Farrar, Valentina Piano, Sebastian Zetzsche, Nico Fuhrmann, Jutta Becker, Mert Karakaya, Yougang Zhang, Yuqing Cao, Afsaneh Taghipour-Sheshdeh, Brett W. Stringer, Jean Giacomotto

**Affiliations:** 1 Institute of Human Genetics, University of Cologne, Faculty of Medicine, Cologne, Germany; 2 Center for Molecular Medicine Cologne, University of Cologne, Cologne, Germany; 3 Center for Rare Diseases, University Hospital of Cologne, University of Cologne, Cologne, Germany; 4 Institute for Biomedicine and Glycomics, Griffith University, Brisbane, QLD 4111, Australia; 5 Department of Pediatric Neurology, Centre for Neuromuscular Disorders, University Hospital Essen, Essen, Germany; 6 Women’s Health, Paediatrics and Child Health, School of Clinical Medicine, University of New South Wales, Sydney, NSW 2033, Australia; 7 Department of Neurology, Sydney Children’s Hospital, Randwick, NSW 2031, Australia; 8 School of Environment and Science, Griffith University, Brisbane, QLD 4111, Australia; 9 Thompson Institute, National PTSD Research Centre, University of the Sunshine Coast, Birtinya, QLD 4575, Australia; 10 Queensland Brain Institute, The University of Queensland, Brisbane, QLD 4067, Australia

**Keywords:** spinal muscular atrophy, *SMN1*, newborn screening, variant interpretation, functional studies, zebrafish model, false positive genetic, *SMN2*, genetic epidemiology, thermostability assay

## Abstract

Newborn screening (NBS) for spinal muscular atrophy (SMA) enables rapid diagnosis and pre-symptomatic treatment of infants with bi-allelic *SMN1* deletions. Standard PCR-based assays detect ∼95% of cases by identifying the absence of *SMN1* exon 7; however, rare sequence variants can escape detection. We describe two newborns (in Germany and Australia) identified by NBS as lacking *SMN1* but subsequently shown to carry a single *SMN1* copy—with no *SMN2* in P1 and one *SMN2* copy in P2. Gene-specific long-range PCR and Sanger sequencing revealed two distinct 4-bp deletions in *SMN1* exon 7 (c.855_858delAGAA [p.Arg288AlafsTer5] in P1 and c.861_864delAAGG [p.Arg288AlafsTer5] in P2). Both variants disrupt the reverse primer-binding site used in NBS assays and cause the same frameshift p.Arg288AlafsTer5, predicted to be deleterious. A plethora of assays demonstrated preserved exon 7 splicing, markedly reduced SMN protein abundance, and wild-type-like protein thermostability. *In vivo*, expression of the p.Arg288AlafsTer5 protein in zebrafish fully rescued the progressive motor and survival defects of *smn1*-deficient mutants. These findings raise the possibility that this novel SMN isoform has enhanced functional efficiency relative to the wild type. Population data (gnomAD) suggest that ∼800 individuals of European ancestry may carry these variants in *trans* with an *SMN1* deletion, yet none have been reported with SMA. Based on our data, no therapy was initiated. Both children remain healthy at 24 months of age, avoiding >US$4 million in potential treatment costs. These findings challenge the assumption that complete loss of full-length SMN invariably causes SMA and suggest that very low levels of this novel SMN isoform can sustain normal motor development.

## Graphical abstract

**Figure:**
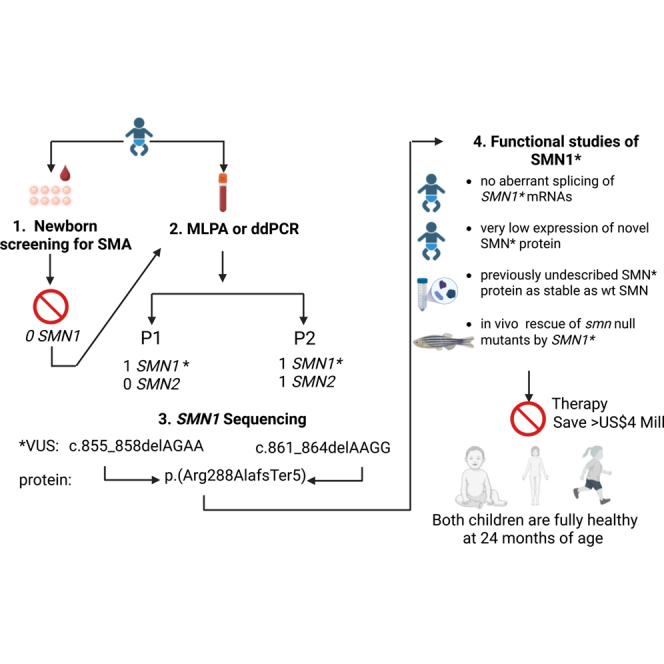

## Teaser

Two different 4-base-pair deletions in *SMN1* that cause a similar frameshift led to false-positive SMA newborn screening results. Integrating genomic, functional, and population data clarified diagnosis, preventing unnecessary treatment, and demonstrated that a previously undescribed SMN protein can preserve motor function despite the absence of wild-type full-length SMN.

## Main text

Spinal muscular atrophy (SMA) is a common autosomal-recessive neuromuscular disorder characterized by progressive degeneration of motor neurons, leading to muscle atrophy. Approximately half of all individuals with bi-allelic *SMN1* (MIM: 600354) loss-of-function (LoF) variants, if left untreated, develop severe SMA type I (MIM: 253300), which is characterized by early death and an inability to sit or walk. The remaining individuals present with intermediate SMA type II (MIM: 253550), in which affected persons are able to sit but not walk, or with milder forms such as SMA type III (MIM: 253400) and, more rarely, adult-onset SMA type IV (MIM: 271150), in which affected persons can sit and walk but often become wheelchair bound as the disease progresses.^1^ SMA is caused by bi-allelic loss or mutated survival motor neuron 1 (*SMN1*), while disease severity is largely influenced by *SMN2* (MIM: 601627), a nearly identical copy gene (http://www.omim.org). The *SMN2* copy number varies from 0 to 4 per allele, with higher copy numbers generally conferring milder phenotypes.^2^ Most *SMN2* transcripts are alternatively spliced, excluding exon 7, and produce a truncated, unstable protein. Only about 10% of *SMN2* transcripts are full-length, encoding a protein identical to *SMN1*.^3^^,^^4^

The recent development of highly effective therapies targeting *SMN1* gene replacement or *SMN2* splicing correction has transformed SMA treatment and management.^5^ Newborn screening (NBS) for SMA is now widely implemented,^6^ including in Germany^7^ and Australia,^8^ enabling early diagnosis and prompt initiation of therapy, which significantly improves outcomes.^9^^,^^10^ The majority of individuals affected by SMA (∼95%) are identified by PCR-based assays detecting bi-allelic *SMN1* exon 7 deletions or gene conversions of *SMN1* into *SMN2*.^11^^,^^12^^,^^13^^,^^14^ However, 3%–5% of individuals with SMA harbor rare sequence variants in *SMN1* that are not detected by standard NBS assays.^2^^,^^15^^,^^16^

**Figure 1:**
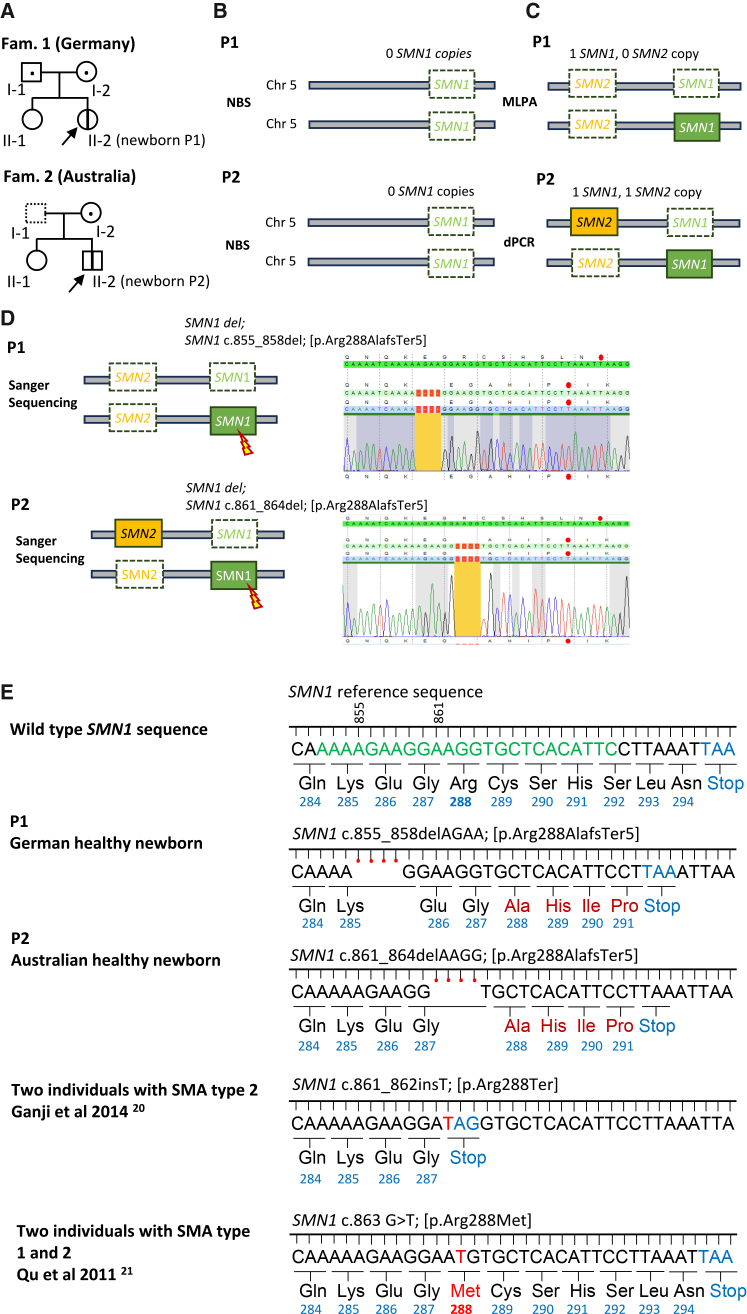
Identification and genetic analysis of two *SMN1*-VUSs in newborns (A) Pedigrees of the two newborns, P1 and P2. Both were asymptomatic at birth and remained asymptomatic at 2 years of age (indicated by a thick vertical line). The older sisters (II-1) of each newborn were unaffected (open circle). The parents of P1 were carriers (dotted symbols). For P2, the father was unavailable for testing (dashed square), and the mother was a carrier (dotted circle). (B) Schematic representation of *SMN1* newborn genetic testing using DNA from dried blood spots (DBSs). No *SMN1* signal (blank rectangle) was detected in the *SMN1* exon 7 PCR in either newborn: P1 (from Germany) or P2 (from Australia). For P1, the LightMix TREC SMA HBB kit (Roche) was used; for P2, the Eonis SCID-SMA kit (3234-0010) was employed. (C) Schematic representation of confirmatory testing using the MRC-Holland P021 MLPA kit (P1) and multiplexing droplet digital PCR (ddPCR) for P2. Both newborns showed one *SMN1* copy (green rectangle). P2 had one *SMN2* copy (orange rectangle), while P1 had none (blank rectangle). (D) Schematic representation of the SMA alleles in both newborns. A chromatogram of Sanger sequencing shows the *SMN1*-VUS (orange block). Deleted nucleotides are marked by small red rectangles. The entire genomic region spanning the *SMN1* gene was amplified via long-range PCR (exons 1–8)^19^ and used as a template to sequence exon 7. A premature stop codon (red dot) is shown in the VUS allele compared to the reference *SMN1* sequence. (E) Schematic reference *SMN1* exon 7 sequence. NBS primers are indicated in green. Both *SMN1*-VUSs (4-bp deletion, red dots), altered the C-terminal amino acid sequence (red letters), and premature stop codons are shown. Two additional pathogenic variants from individuals with SMA are included for comparison.

Here, we report two newborns identified by NBS as lacking both *SMN1* copies: one from Germany (individual II-2 from family 1; P1) and one from Australia (individual II-2 from family 2; P2) (Figures 1A and 1B). Instead, confirmatory testing by multiplex ligation-dependent probe amplification (MLPA; MRC-Holland kit P21-B1^17^) or multiplexing droplet digital PCR (ddPCR)^18^ revealed one *SMN1* in both infants, with zero *SMN2* in P1 and one *SMN2* in P2 (Figure 1C). Long-range PCR of genomic *SMN1*^19^ followed by Sanger sequencing identified distinct variants of uncertain significance (VUSs) in exon 7 in each newborn: in P1, a 4-bp deletion was detected (*SMN1*: c.855_858delAGAA [GenBank: NM_000344.4] [p.Arg288AlafsTer5], abbreviated 855VUS). In P2, a different 4-bp deletion was identified (*SMN1*: c.861_864delAAGG [GenBank: NM_000344.4] [p.Arg288AlafsTer5], abbreviated 861VUS) (Figure 1D). No further variants were detected in the remaining *SMN1* exons. Both deletions overlap the NBS assay’s reverse primer-binding site, explaining the absence of an *SMN1* result (Figure 1E). Haplotype analysis showed that the c.855_858delAGAA variant in P1 was paternally inherited, while the maternal allele carried a deletion. In P2, only the mother was available, and she was a carrier of the *SMN1* deletion. P2 has one older sister, who does not carry the *SMN1* variant allele; no further relatives were available for this study (Figure 1A).

Both *SMN1* variants result in the same frameshift mutation: p.Arg288AlafsTer5, altering the C-terminal region and introducing a premature stop codon (Figure 1E). These variants were classified by both genetic labs as VUSs. Possible pathogenicity was initially supported by prior reports of pathogenic mutations near the same region in individuals with SMA: two Iranian individuals with SMA type II with a 1-bp insertion (c.861_862insT [p.Arg288Ter])^20^ and two Chinese individuals with SMA types I and II carrying a missense variant (c.863G>T [p.Arg288Met]), which caused complete *SMN1* exon 7 skipping (Figure 1D).^21^

The identification of these *SMN1* variants in two clinically healthy newborns presented a major clinical and diagnostic dilemma. Most striking was the observation that P1, who has no *SMN2* copies and hence no known source of wild-type (WT) SMN protein, remained completely asymptomatic. It has long been assumed—supported by mouse models—that a minimum amount of WT, full-length SMN protein is required for survival.^22^^,^^23^ Therefore, a proband such as P1 with no functional *SMN1* and no *SMN2* copies would result in a miscarriage or stillbirth. P1’s asymptomatic state essentially proved that this altered *SMN1* variant has at least some residual function.

**Figure 2:**
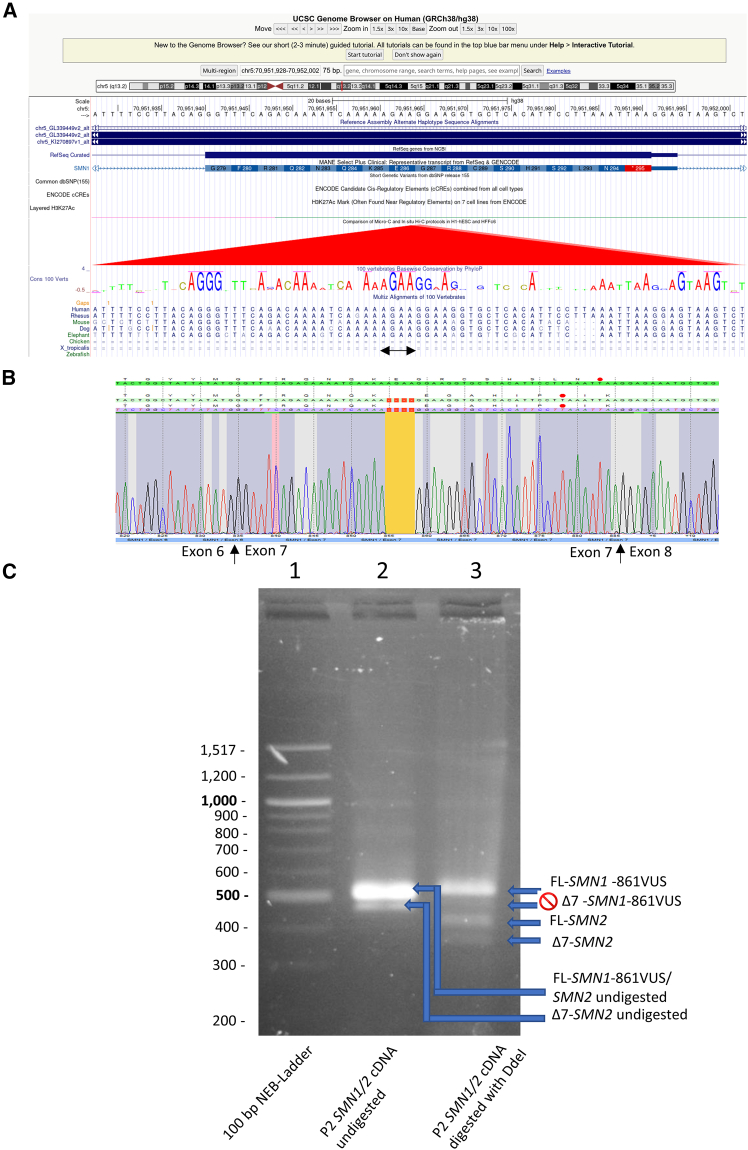
Splicing of *SMN1*-VUS in patient-derived lymphoblastoid cell lines is unaffected (A) The RNA region chr5:70951940–70951980 (GRCh38/hg38) encompassing the two *SMN1*-VUSs overlaps with a significant Hi-C peak in ENCODE data, suggesting potential regulatory relevance (https://genome.ucsc.edu). (B) Chromatogram of *SMN1-855VUS* cDNA showing clean exon-exon boundaries between exons 6–7 and 7–8, with no evidence of exon 7 skipping in P1, ruling out aberrant splicing. cDNA was reverse transcribed from total RNA isolated from the Epstein Barr Virus (EBV)-transformed lymphoblastoid cell line of P1 and *SMN1-855VUS* transcripts amplified with primers localized in exons 5 and 8. (C) PCR analysis of *SMN* transcripts (exons 5–8) after DdeI digestion in P2. For *SMN1-861VUS*, only full-length (FL) transcripts are visible; no Δ7-*SMN1*-861VUS transcripts are detected. In contrast, *SMN2* yields both FL and Δ7 transcripts. cDNA was reverse transcribed from total RNA isolated from the EBV-transformed lymphoblastoid cell line of P2.

Given that the p.Arg288Met mutation was previously shown to severely affect splicing,^21^ we investigated the local chromatin regulatory landscape provided by ENCODE. Hi-C data indicate a strong *cis*-regulatory element in this region (Figure 2A). We established lymphoblastoid cell lines from both probands to assess *SMN1* splicing. Sanger sequencing of exon 5–8 cDNA amplicons from P1’s RNA revealed a clear sequence of full-length *SMN1*, excluding alternatively spliced transcripts, and provided additional evidence that only *SMN1* is present in this newborn (Figure 2B). In P2, *SMN1* and *SMN2* transcripts were distinguished by DdeI digestion, which cuts specifically in *SMN2* exon 8.^3^ *SMN1* transcripts from the variant allele were only full-length, indicating no impact on splicing (Figure 2C).

**Figure 3:**
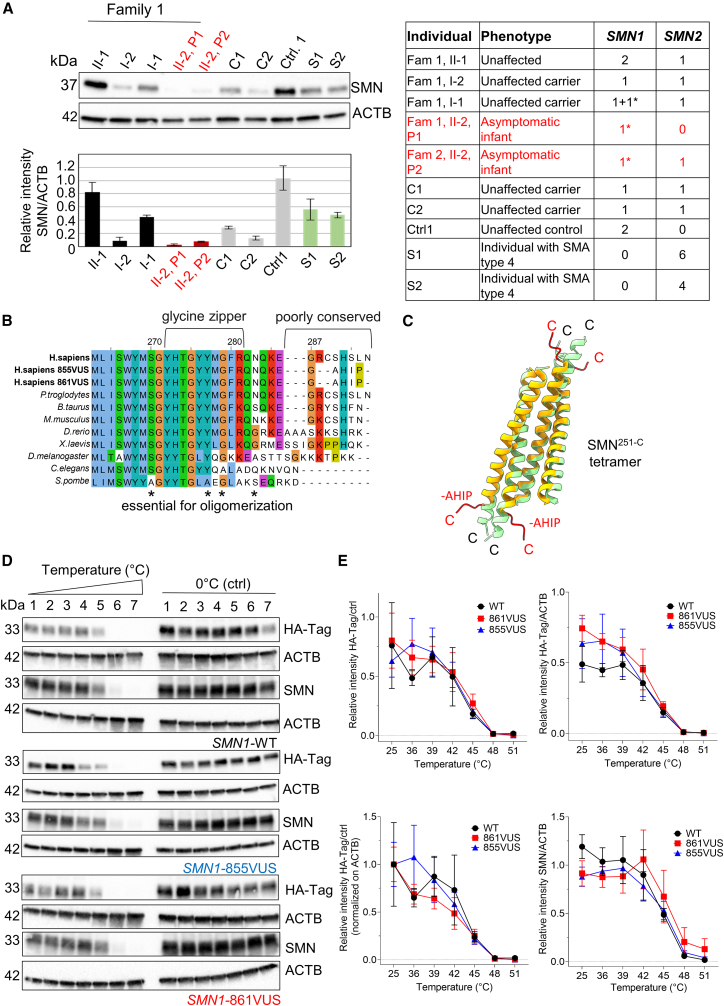
SMN protein abundance is low in newborns, but SMN-VUS proteins are thermostable (A) Representative western blot and quantification of protein lysates from EBV-transformed lymphoblastoid cell lines were performed as previously described.^24^^,^^25^ SMN and actin (ACTB, loading control) levels are shown for P1 and P2, P1 family members, carriers, control, and individuals with SMA type IV. SMN/ACTB ratios are presented as mean ± SD (*n* = 4). Monoclonal antibodies against SMN (BD Biosciences) and ACTB (Proteintech) were used. The corresponding phenotype and *SMN1/SMN2* genotype for each individual are provided in the table on the right. (B) C-terminal amino acid alignment of SMN across species, highlighting high conservation in the glycine zipper and oligomerization domain (red arrows) and lower conservation in the region containing the SMN-VUS. (C) AlphaFold3 structural predictions show that the SMN-VUS (orange) has a shorter helix and mildly altered tetramerization compared to SMN wild-type (WT) in green. (D) Thermostability assay of HA-tagged *SMN1*-WT, HA-*SMN1*-855VUS, and HA-*SMN1*-861VUS expression constructs, transfected in HeLa cells. Protein lysates were prepared after heating from 36°C to 51°C or kept on ice (control). Actin (ACTB), a well-known thermostable protein, was used as a reference. Western blot and immunostaining were performed using HA (Jackson ImmunoResearch), SMN (BD Biosciences), and ACTB (Proteintech) antibodies. (E) Quantifications of the Western blots included HA/control, HA/ACTB, HA/control/ACTB, and SMN/ACTB. SEM from four independent experiments showed no significant differences between SMN-WT and SMN-VUS proteins.

Western blot analysis of lymphoblastoid cell lysates from P1 and P2, family members of P1, individuals with SMA type IV, SMA carriers, and control subjects showed that both P1 and P2 produced extremely low SMN protein levels—lower than all other samples tested (Figure 3A; supplemental information). Despite this, both children remained clinically well.

Despite the frameshift altering the C-terminal region (residues 282–295), the SMN VUS preserves the glycine and histidine residues that are well conserved in mammals and suggest a potential mammalian-specific function (Figure 3B). To predict the impact of the mutation on SMN 3D structure and stability, we used AlphaFold3^26^ (https://alphafoldserver.com) and input the SMN C-terminal region as homo-tetramer or octamer (Figures 3B and S2; supplemental information).^27^^,^^28^ The α helix oligomerization domain^29^ (Figure 3B) is mildly altered by the p.Arg288AlafsTer5 variant but not by p.Arg288Ter (Figure 3C).

We then expressed HA-tagged constructs of WT *SMN1-WT*, or mutated *SMN1*-855VUS, and *SMN1*-861VUS in HeLa cells and assessed thermostability by exposing lysates to increasing temperatures (36°C–51°C). All three proteins showed similar thermostability, comparable to actin, a protein well-known for its thermostability,^30^ suggesting that the mutated SMN proteins were structurally stable (Figure 3D; supplemental information).

Using support from the Australian Functional Genomics Network, we rapidly adapted a zebrafish model to evaluate the *in vivo* functionality of *SMN1* variants.^31^ In this pilot methodological study, bi-allelic *smn1* LoF zebrafish^32^ were injected with a range of control and variant mRNA.^31^ Known pathogenic *SMN1* variants failed to rescue the *smn1* LoF phenotype. In contrast, the *SMN1*-855VUS and *SMN1*-861VUS mRNA variants fully reproduced the functional complementation obtained with WT or known benign variant mRNAs. Functional complementation was assessed through normal morphology, swimming behavior, and survival during the first week of life, until the transiently injected mRNA and protein were degraded.^31^

**Figure 4:**
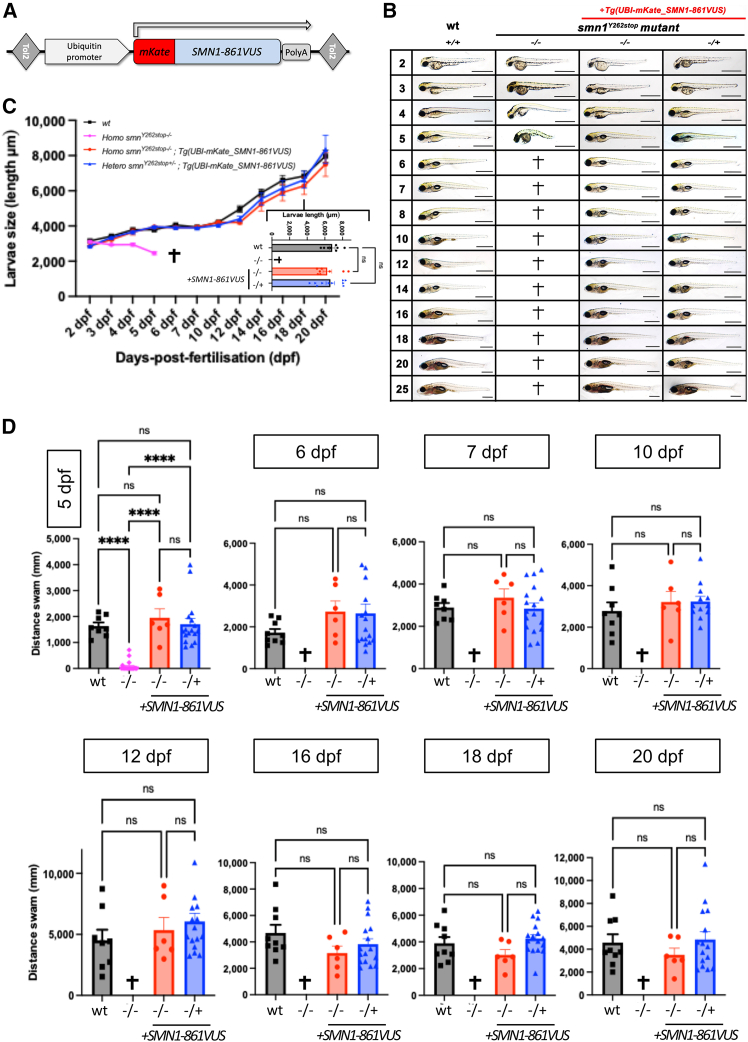
*SMN1-861VUS* transgene fully complements the loss of SMN function in zebrafish (A) DNA transgene *Tg*(*UBI-mKate_SMN1-861VUS*) integrated into the genome of zebrafish mutants *smn1*^Y262stop−/+^. (B) Representative images showing morphology from 2 to 25 days post-fertilization (dpf). Homozygous *smn1*^Y262stop−/−^ mutants exhibit progressive deterioration and premature death between 5 and 6 dpf, while expression of *Tg*(*UBI-mKate*_*2*_*-SMN1-861VUS*) restores normal development, morphology, and survival. Scale bar: 1,000 μm. (C) Growth curves showing larval length measurements (±SEM) from 2 to 20 dpf. No significant difference was observed between wild-type (WT), heterozygous, and *Tg*(*UBI-mKate_SMN1-861VUS*)-complemented *smn1*^Y262stop−/−^ animals. The inset (bottom right) highlights comparable body size at 18 dpf. (D) Motor function comparison from 5 to 20 dpf. Total distance swam (±SEM) during a 24-min recording. Homozygous *smn1*^Y262stop−/−^ mutants display rapid motor function loss culminating in near paralysis by 5 dpf, whereas the transgene *Tg*(*UBI-mKate_SMN1-861VUS*) restores normal locomotor activity indistinguishable from WT and heterozygous controls. Data were analyzed using a non-parametric Kruskal-Wallis test, followed by Dunn’s post hoc correction for multiple comparisons; ^∗∗∗∗^*p* < 0.0001.

To complement this initial transient mRNA supplementation study, here, we generated a stable transgenic line, *Tg*(*UBI-mKate*_*2*_*-SMN1-861VUS*), ubiquitously expressing *SMN1*-861VUS. The transgene was integrated into the genome of *smn1*^Y262stop−/+^ zebrafish mutants (Figure 4A; supplemental information; Video S1) using Tol2-mediated transgenesis.^33^ As previously shown, all homozygous *smn1*^Y262stop−/−^ mutants exhibited progressive loss of motor function and died before 6 days post-fertilization (dpf).^31^ In contrast, the presence of the transgene encoding the p.Arg288AlafsTer5 protein restored normal development, morphology, and motor function, indistinguishable from WT and heterozygous controls (Figures 4B and 4C), confirming that the encoded protein is functional.

Video S1. Representative video recordings of 20 dpf WT control vs. homozygote *smn1*^Y262stop−/−^*; Tg*(*UBI-mKate_SMN1-861VUS*) and heterozygote *smn1*^Y262stop+/−^*; Tg*(*UBI-mKate_SMN1-861VUS*) Videos acquired using the Zebrabox revolution, 24-min recordings comprising three cycles of 4 min of light and 4 min of dark. The videos were assembled and annotated in Adobe Premiere Pro. All homozygous negative controls *smn1*^Y262stop−/−^ died at 5 dpf.

Population-level data from gnomAD v.4.1 (806,504 genomes/exomes) showed that both variants are rare and found exclusively in individuals of European ancestry (*N* = 589,724). The c.855_858delAGAA variant was observed 13 times and the c.861_864delAAGG variant 47 times, all in a heterozygous state (https://gnomad.broadinstitute.org). No homozygotes were reported. This results in a combined carrier frequency of ∼1 in 9,828 individuals.

Given an *SMN1* deletion carrier frequency of ∼1 in 35 in Europeans,^34^ the expected frequency of compound heterozygotes (*SMN1* deletion: *SMN1*-855VUS or *SMN1*-861VUS) is ∼1 in 1.37 million, assuming autosomal-recessive inheritance and random mating (1/35 × 1/9,828 × 1/4 = 1/1.37 million). Based on a current estimated world population of approximately 8.2 billion, with ∼13.88% (or ∼1.13 billion individuals) of European descent, ∼800 (1.13 billion/1.37 million = 824) individuals are predicted to exist who harbor the compound heterozygous genotype—yet none have been reported with SMA, further supporting non-pathogenicity.

The almost simultaneous discovery of the two variants in Germany and Australia aligns with expectations from current NBS coverage. In 2024, ∼64% of newborns in the European Union 27 (EU27) were screened for SMA (https://www.sma-europe.eu/newborn-screening-in-sma). In 2023, 3.67 million children were born in the EU27 (https://ec.europa.eu/eurostat/statistics-explained/index.php?title=Fertility_statistics), suggesting that ∼2–3 children per year should be compound heterozygous for an *SMN1* deletion with either 855VUS or 861VUS (3.67 million/1.37 million ≈ 2.6).

Additional rare missense variants in primer or probe-binding regions reported in gnomAD v.4.1 (Table S1; Figure S1), ClinVar (Table S2), and NBS programs^11^^,^^35^^,^^36^^,^^37^ may lead to even more false-positive or uncertain NBS results. Considering all 15 reported variants reported in gnomAD within the annealing primer region, of which 9 occur in Europeans, 229 alleles in 227 individuals (112 alleles in 111 Europeans) were identified. This would increase the estimated number of compound heterozygotes in Europeans to ∼1,500 (1.13 billion/743,796 ≈ 1,519), with ∼5 expected newborns per year in the EU27. Indeed, in both countries, besides the newborns reported here, an additional variant was identified in the initial NBS screening program in Australia,^11^ and thus far, the proportion of true-positive results is 96.3%. We are aware of an additional false-positive case in Germany that has not yet been published.

To increase diagnostic accuracy, we recommend that any abnormal result in SMA NBS (bi-allelic absence of *SMN1*) that cannot be confirmed via MLPA or ddPCR should always be followed by complete *SMN1* sequencing, as a known or novel variant within the annealing primer site is the most likely explanation. This combined diagnostic strategy is already implemented in the Australian SMA NBS guidelines (https://doi.org/10.26190/unsworks/31305) and could be adopted globally in such cases.

The strong rescue studies in zebrafish^31^ and this study, together with the functional data presented here, as well as the population data, argue that these two VUS alleles are likely non-pathogenic. This assumption is further supported by the analysis of hypomorphic *SMN1* alleles in SMA zebrafish, which—contrary to both *SMN1* VUSs—are unable to fully rescue survival.^31^ We speculate that the novel SMN protein retains function and may even possess gain-of-function properties, as it supports survival despite very low abundance.

For P1, gene therapy with Zolgensma was the only potential therapeutic option, as no *SMN2* copies were present. However, since the child already expressed a previously undescribed functional SMN protein and lacked prior exposure to WT SMN, the risk of an immune response was uncertain. For P2, gene therapy with Zolgensma or splice-modifier therapy with nusinersen or risdiplam would have been an option but with limited efficacy expected for the latter, given only one *SMN2* copy.^9^^,^^38^^,^^39^

Ultimately, after reviewing all functional and epidemiological evidence, neither child was started on therapy. Now, at 24 months of age (at the date of submission of the manuscript), both children show normal motor development, confirmed by regular clinical and electrophysiological assessments (supplemental notes). Forgoing treatment avoided patient stress and saved healthcare costs exceeding US $4 million.

In conclusion, our integrated functional, structural, and population-level analyses support a likely non-pathogenic reclassification for the *SMN1* c.855_858delAGAA and c.861_864delAAGG variants. These findings directly influenced clinical decision-making, enabling appropriate care while avoiding unnecessary and costly therapies. Notably, we describe a unique scenario in which very low levels of an altered SMN protein appear to preserve normal motor function despite a complete absence of WT SMN—a condition previously thought incompatible with survival. Detailed functional and cellular studies of this altered protein in the future may uncover enhanced or modified properties and interaction networks, which may be of therapeutic relevance.

## Data and code availability

Data generated or analyzed during this study are included in the published article and the corresponding supplemental information. Zebrafish resources used in this study include the previously described *smn1*^*Y262stop*^ mutant line and a newly generated transgenic line, *Tg*(*UBI-mKate*_*2*_*-SMN1-861VUS*). These lines are not deposited in public repositories but are available from Dr. J. Giacomotto upon reasonable request, in accordance with institutional and ethical regulations.

## Acknowledgments

We are grateful to both families. This work was funded as follows: Australian Functional Genomics Network Catalyst Grant #11501 to J.G., S.G., and B.W.; a Center for Molecular Medicine Cologne (CMMC) grant to BW (C18); and NHMRC fellowship no. 1174145 to J.G. The Australian Functional Genomics Network is funded by the Medical Research Future Fund (funding ID MRF2007498) and administered by the Murdoch Children’s Research Institute.

## Author contributions

Conceptualization, B.W. and J.G.; data acquisition, J.D., S.Z., Y.Z., Y.C., A.T.-S., and B.W.S.; data analysis, B.W., J.D., V.P., N.F., J.B., S.Z., H.K., M.A.F., S.G., M.K., and J.G.; funding acquisition, J.G., S.G., and B.W.; visualization, B.W., V.P., and J.G.; clinical data interpretation, H.K., M.A.F., S.G., and M.K.; writing – original draft, B.W.; writing – review and editing, all authors. All co-authors read and approved the final manuscript.

## Declaration of interests

The authors declare no competing interests.

## Web resources

AlphaFold3, https://alphafoldserver.com
Ensembl, https://www.ensembl.org
Eurostat, https://ec.europa.eu/eurostat/statistics-explained/index.php?title=Fertility_statistics
gnomAD, https://gnomad.broadinstitute.org
OMIM, http://www.omim.org
SMA Europe, https://www.sma-europe.eu/newborn-screening-in-sma
UCSC Genome Browser, https://genome.ucsc.edu

## Supplemental information

Document S1. Figures S1 and S2, Tables S1 and S2, supplemental notes, and supplemental methods

Document S2. Article plus supplemental information
