# Supplementary material for: SMN1 variants identified by false-positive SMA newborn screening tests: Therapeutic hurdles and functional and epidemiological solutions
Source: Am J Hum Genet. 2026 Feb 12;113(3):627–35. doi: 10.1016/j.ajhg.2026.01.012 (PMC13087420; doi:10.1016/j.ajhg.2026.01.012)
Supplement: Document S1. Figures S1 and S2, Tables S1 and S2, supplemental notes, and supplemental methods [file mmc1.pdf]

The American Journal of Human Genetics, Volume *113*

Supplemental information

SMN1 variants identified by false-positive SMA newborn screening tests: Therapeutic hurdles and functional and epidemiological solutions

Brunhilde Wirth, Joyosmita Das, Heike Kölbel, Shuxiang Goh, Michelle A. Farrar, Valentina Piano, Sebastian Zetzsche, Nico Fuhrmann, Jutta Becker, Mert Karakaya, Yougang Zhang, Yuqing Cao, Afsaneh Taghipour-Sheshdeh, Brett W. Stringer, and Jean Giacomotto

Supplemental Figure, Tables, Notes and Methods

Figure S1. *SMN1* sequence showing all variants identified in GnomAD in the *SMN1* reverse primer annealing region.

The reverse *SMN1* primer sequence used for NBS testing is depicted in green. Shown are the SNPs and 4-bp deletion variants as well as their frequency in GnomAD. The resulting amino acid exchanges are given in the lower part. The ID accession no: NM_000344.4 (*SMN1*)

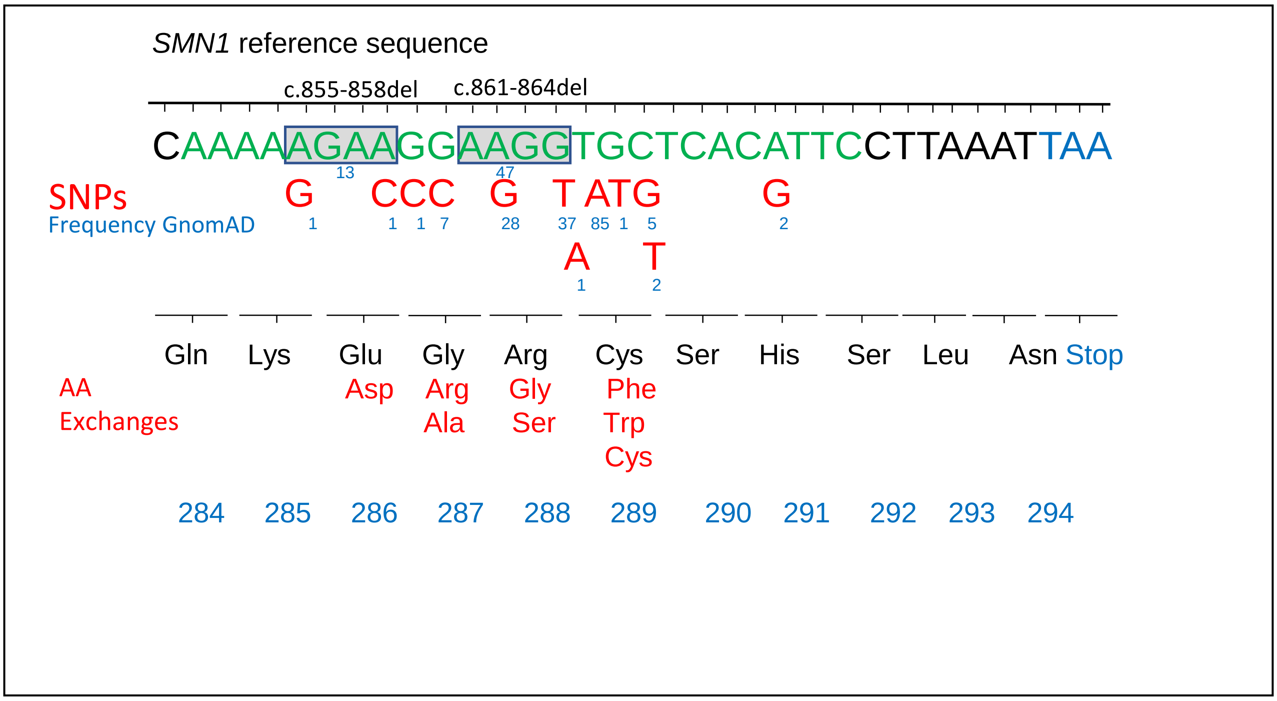

Figure S2. Predicted aligned error (PAE) plots for the SMN WT and SMN1 VUS tetramer and octamer models.

A. PAE plot of the AF3 model of SMN WT tetramer. B. PAE plot of the AF3 model of SMN1 VUS tetramer. C. PAE plot of the AF3 model of SMN WT octamer. D. PAE plot of the AF3 model of SMN1 VUS octamer.

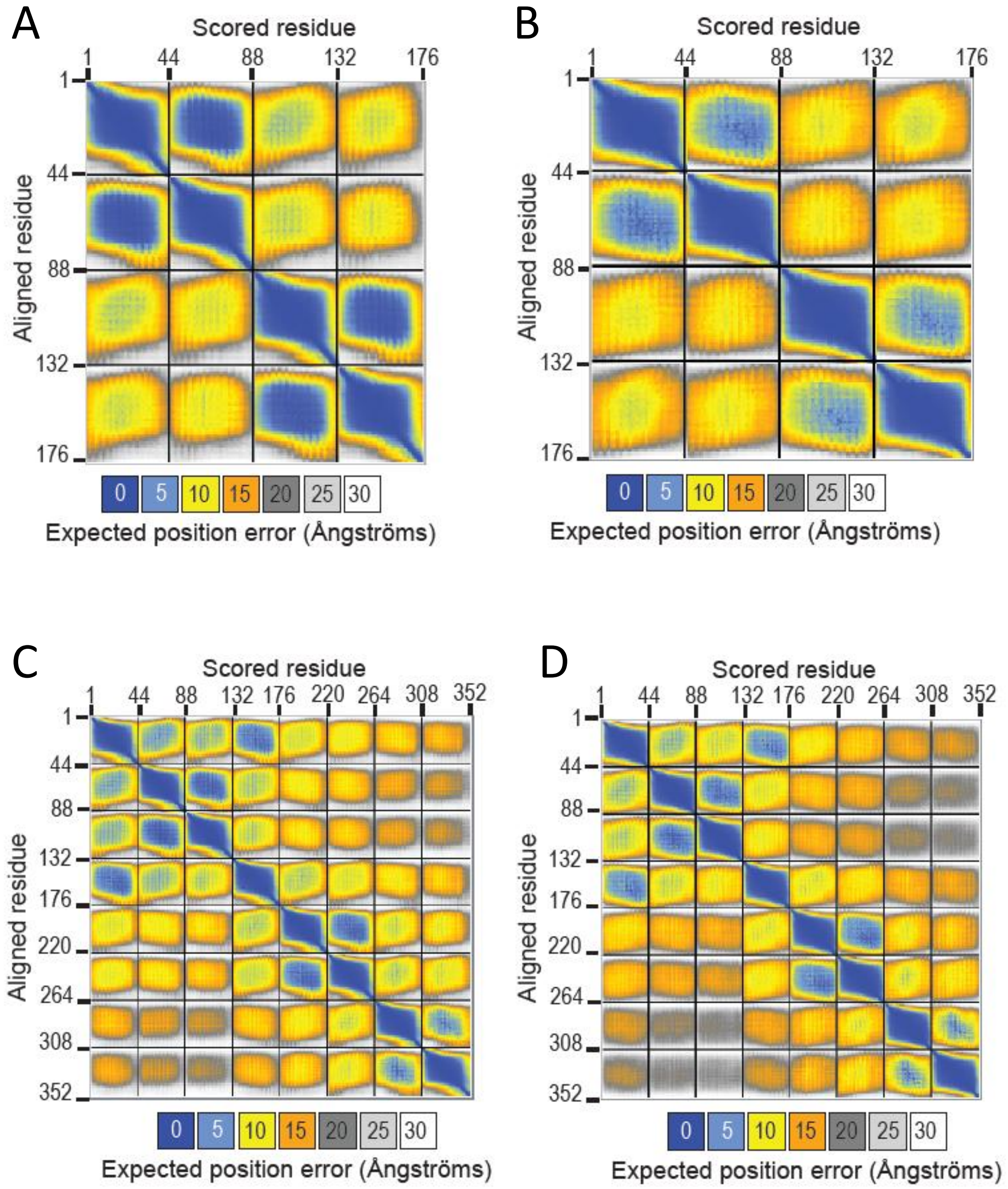

Table S1. Variants reported in gnomAD v4.1 corresponding to the reverse *SMN* primer sequence (chr5:70,951,957-70,951,981) used for NBS testing.

Variant ID = ID in SNP database; Source E= exome, G= genome, HGVS consequence = HGVS protein sequences; VEP=Variant effect predictor; Germline classification= ClinVar germline classification; Allele count is given for all tested E/G and in brackets the counts in the population of European origin. The ID accession no: NM_000344.4 (*SMN1*)

Variant ID	Source	HGVS Consequence	VEP annotation	Germline classification	Allele count (EU)		Allele Number	Allele Frequency	No of homozygous
5-70951957-A-C	E	p.Gln284Pro	missense		3	(3)	1613152	1.86e-6	0
5-70951958-AAAAG-A	G	p.Arg288AlafsTer5	frameshift	Confl. classif. of pathog.	13	(13)	1613162	1.10e-5	0
5-70951961-A-G	E	p.Lys285Lys	synonymous		1	(0)	1613130	6.20e-7	0
5-70951961-AGAAG-A	G	p.Arg288AlafsTer5	frameshift	Confl. classif. of pathog.	47	(47)	1613008	2.91e-5	0
5-70951964-A-C	E	p.Glu286Asp	missense		1	(1)	1613094	6.20e-7	0
5-70951965-G-C	G	p.Gly287Arg	missense	Uncertain significance	1	(0)	1612830	6.20e-7	0
5-70951966-G-C	E	p.Gly287Ala	missense		7	(7)	1613004	4.34e-6	0
5-70951968-A-G	EG	p.Arg288Gly	missense	Uncertain significance	28	(27)	1613040	1.74e-5	1
5-70951970-G-T	EG	p.Arg288Ser	missense	Uncertain significance	37	(0)	1612796	2.29e-5	0
5-70951970-G-A	E	p.Arg288Arg	synonymous		1	(0)	1612918	6.20e-7	0
5-70951971-T-A	EG	p.Cys289Ser	missense	Uncertain significance	85	(9)	1613186	5.27e-5	1
5-70951972-G-T	E	p.Cys289Phe	missense		1	(0)	1613046	6.20e-7	0
5-70951973-C-G	G	p.Cys289Trp	missense		5	(3)	1612820	3.10e-6	0
5-70951973-C-T	G	p.Cys289Cys	synonymous		2	(0)	1612820	1.24e-6	0
5-70951978-A-G	E	p.His291Arg	missense		2	(2)	1613088	1.24e-6	0
**Sum**					229		~1613000	7.04e-4	2
						(112)	1179448	1.05e-5	1

https://genome.ucsc.edu/cgi-bin/hgTracks?db=hg38&lastVirtModeType=default&lastVirtModeExtraState=&virtModeType=default&virtMode=0&nonVirtPosition=&position=chr5%3A70951932%2D70952002&hgsid=2907429012_iAmAMNyWRCnXOiJckcXRmlHPeN0k

Table S2. Variants reported in ClinVar corresponding to the reverse *SMN1* primer sequence (chr5:70,951,957-70,951,981) used for NBS testing.

Link to ClinVar with Variant ID	Molecular consequence	dbSNP ID	no of submitter	clinical significance
VCV000495832 : NM_000344.4(*SMN1*):c.855dup (p.Glu286fs)	frameshift	rs1554082383	1	VUS
VCV001256472 : NM_000344.4(*SMN1):c.855_858del (p.Arg288fs)*	frameshift	rs1475940018	2	VUS
VCV002190592 : NM_000344.4(*SMN1*):c.861_864del (p.Arg288fs)	frameshift	rs1179910122	3	Confl. classif. of path.
VCV003339588 : NM_000344.4(*SMN1*):c.859G>C (p.Gly287Arg)	missense		1	VUS
VCV000644259 : NM_000344.4(*SMN1*):c.862dup (p.Arg288fs)	frameshift	rs1580895068	2	SMA
VCV001949916 : NM_000344.4(*SMN1*):c.862A>G (p.Arg288Gly)	missense	rs1472645065	1	VUS
VCV002988035 : NM_000344.4(*SMN1*):c.862A>T (p.Arg288Trp)	missense	rs1472645065	2	VUS
VCV000928625 : NM_000344.4(*SMN1*):c.864G>T (p.Arg288Ser)	missense	rs368899583	2	VUS
VCV000495833 : NM_000344.4(*SMN1*):c.865T>A (p.Cys289Ser)	missense	rs187925143	2	VUS
VCV000634943 : NM_000344.4(*SMN1*):c.866G>A (p.Cys289Tyr)	missense	rs765273240	1	VUS
VCV003896022 : NM_000344.4(*SMN1*):c.872dup (p.His291fs)	frameshift		1	VUS
VCV000634944 : NM_000344.4(*SMN1*):c.873T>A (p.His291Gln)	missense	rs1561503207	2	VUS

Supplemental Note: Case Reports

P1 (II-2, Family 1) is the second child of non-consanguineous parents from their second pregnancy. The older daughter is healthy and exhibits no developmental concerns. The pregnancy with P1 progressed without complications, with no reported infections and normal fetal movements. Postpartum, following a cesarean section due to breech presentation, she displayed no signs of adjustment disorders, achieving an APGAR score of 10/10. During her initial assessment at 2 weeks of age, she obtained a score of 63 out of 66 on the Children's Hospital of Philadelphia Infant Test of Neuromuscular Disorders (CHOP INTENT), indicating developmentally appropriate performance. By 3 months, she achieved a perfect score of 66 out of 66 in subsequent evaluations. At 9 months, she demonstrated secure alternating crawling, independent sitting, standing with full weight-bearing on her legs, and taking lateral steps. By 12 months, she was able to walk independently. Now, at 24 months of age, she displays normal motor skills, including running, jumping, and climbing. Her language and cognitive development, assessed using the Bayley III scales, also yielded normal results. In the most recent physical examination at 24 months, her muscle tone was normal, with no contractures or signs of muscle atrophy. Deep tendon reflexes were symmetrical, and there were no tongue fasciculations. She demonstrated no difficulties in rising from the ground or climbing stairs. Neurophysiological examinations conducted at all assessments revealed normal latency and amplitude for the median, ulnar, and tibial nerves.

P2 (II-2, Family 2) is the second child of non-consanguineous parents from their second pregnancy. His 3-year-old sister had no concerns for weakness or neurodevelopment. The pregnancy, delivery at term and postpartum periods were unremarkable. Pediatric examination at 2 weeks of age demonstrated good tone and movements, with no proximal weakness. Deep tendon reflexes were present and symmetrical. He was feeding well with a strong suck reflex and no tongue fasciculations. At 3 months, his CHOP-INTEND score was 62/64 and HINE 8. He was able to roll to either side, had upright head control and sat with support. He had good head control on pull to sit with flexed biceps, kicked his legs vigorously and bore weight when held standing. In ventral suspension he extended his neck, back and legs. His Bayley's gross motor assessment scale was 2 standard deviations above the mean.
He continued to make motor progress and at 8 months, he was able to roll on both sides, sit independently and maintain a 4-point crawling position when placed with extended arms. He was starting to shuffle on his tummy short distances in a commando style, bore weight when held standing and bounced up and down easily. He was walking independently at 14 months of age. Longitudinal Bayley's gross motor assessment scale scores and neurophysiology at ages 8, 14, 20 and 26 months demonstrated increasing scores consistent with neurotypical development. In the most recent assessment, he had normal tone, muscle power, and deep tendon reflexes with no tongue fasciculations. He achieved all WHO motor milestones (minus hands and knees crawling), walked independently and was active, with no fatigue, falls or difficulty getting up from the ground. He was speaking in long phrases and short sentences

Supplemental Methods

Ethical standards

Human ethics approval for this work was granted by the Sydney Children's Hospitals Network Human Research Ethics Committee (approval number 2023/ETH01937) and the Ethics Committee of the University of Cologne (approval number 13-022). Informed written consent for the collection of human material, participation in the study and publication purposes was obtained from the legal guardians of the newborns, in accordance with the regulations of the Ethics Committee of the University of Cologne and Sydney.

Western blot analysis of lymphoblastoid cell lines

Epstein–Barr virus (EBV)–transformed lymphoblastoid cell lines were generated from peripheral blood samples by the Wirth laboratory (P1 and family members, further individuals with SMA, SMA carriers, and controls) and the Farrar laboratory (P2) using standard protocols, as previously described.[38,39] Briefly, peripheral blood samples were collected in EDTA as an anticoagulant (1.5 mg EDTA per mL of blood). A volume of 2.5 mL of blood from pediatric donors or 5 mL from adult donors was used. Blood samples were transferred to 50 mL Falcon tubes and brought to a final volume of 50 mL with sterile erythrocyte lysis buffer (155 mM NH_4Cl, 10 mM $KHCO_3$, and 0.1 mM EDTA, pH 7.4). Samples were incubated for 10 min at room temperature and centrifuged at 1,500 rpm for 10 min. This lysis step was repeated twice. Residual red blood cells at the periphery of the white cell pellet were carefully removed using a pipette tip. The remaining cells were resuspended in 2 mL of EBV-containing supernatant and incubated for 1 h at 37 °C. Subsequently, 10 µL cyclosporine A (1 mg/mL; Merck) and 3 mL of RPMI 1640 medium (Gibco) were added, and the cell suspension was transferred to a T25 culture flask. Cultures were maintained at 37 °C, and successful transformation was monitored daily.

EBV-transformed cell lines were maintained in RPMI 1640 supplemented with 20% fetal bovine serum (FBS), penicillin/streptomycin, and amphotericin B, at 37 °C in 5% CO_2. Medium was replenished every 3–4 days based on color change of the pH indicator (from light red to yellow). The day before harvesting for Western blot analysis, fresh medium was added to allow exponential growth of all cell lines.

Western blot analysis was performed as previously described.[38] Cells were harvested in RIPA buffer (150 mM NaCl, 1% NP-40, 0.5% sodium deoxycholate, 0.1% SDS, 50 mM Tris-HCl, pH 8.0) to prepare total protein extracts. Protein concentration was quantified using a Qubit 3 Fluorometer (Thermo Fisher Scientific) and the Qubit Protein Assay Kit (Q33211, Thermo Fisher Scientific). Denatured protein samples (20 µg per sample) were resolved by 10% SDS–PAGE and transferred onto nitrocellulose membranes using the FastBlot Transfer Kit (Bio-Rad). Protein transfer was assessed by Ponceau S staining (Thermo Fisher Scientific).

Membranes were immunostained and signal detection was performed using a chemiluminescence reagent (SuperSignal™ West Pico PLUS, Pierce, Rockford, USA) according to standard protocols. The following primary antibodies were used: mouse monoclonal anti-actin HRP-conjugated antibody (Proteintech HRP-60008-100UL; 1:5,000) and mouse monoclonal anti-SMN antibody (BD Biosciences 610647;

1:2,000). Antibodies were diluted in 3% non-fat dry milk in TBS-T (20 mM Tris-HCl pH 7.4, 150 mM NaCl, 0.05% Tween-20). Each experiment was repeated at least four times, and band intensities were quantified using Fiji software.

AlphaFold 3 predictions

Protein structure predictions were generated using AlphaFold3 (AF3), employing the standard multimodal pipeline integrating sequence, evolutionary, and structural features. Input protein sequences (SMN1 WT and 855/861VUS) either in 4 or 8 copies were submitted to the AF3 prediction server (AlphaFold Server). The top-ranked model was selected for representation using Chimera X software, and confidence metrics (predicted aligned error, PAE) were used to assess local and global reliability of the predictions (**Figure S2**).

Thermostability assay

HeLa cells were maintained at 37 °C in a humidified atmosphere containing 5% CO_2 and cultured in DMEM supplemented with 10% (v/v) fetal bovine serum (FBS) and 0.1× penicillin–streptomycin. Cells (1×10^5) were seeded into one well of a 6-well plate. After 18 h, the medium was replaced with DMEM/F-12, and cells were transfected with 0.1 µg/mL plasmid DNA. After 48 h, cells were harvested and resuspended in PBS (10 mM Na_2HPO_4, 1.8 mM KH_2PO_4, 137 mM NaCl, 2.7 mM KCl, pH 7.2).
cDNAs of *SMN1* WT, *SMN1* 855VUS and *SMN1* 861VUS were introduced using Gibson cloning assembly in the backbone plasmid AAV Ef1a (Addgene 135428), fusing the HA tag at the N-terminus of SMN1 via PCR (primer_1_F ATACGATGTTCCAGATTACGCTATGGCGATGAGCAGCG, primer_2_F TCGTGAGGTACCATGTACCCATACGATGTTCCAGA)
For each condition, 50 µL of the cell suspension was aliquoted into PCR tubes. Tubes were either kept on ice (0 °C control) or placed in a thermocycler set to 25, 45, 48, 51, 54, 57, or 60 °C. Samples were incubated for 6 min, followed by 6 min at 4 °C. Cells were then centrifuged and lysed in RIPA buffer supplemented with protease inhibitor cocktail (MilliporeSigma). Lysates were incubated on ice for 30 min and centrifuged at 12,000 rcf for 20 min at 4 °C.
A total of 15 µg protein per sample was loaded for SDS–PAGE and Western blotting. Membranes were probed with the following primary antibodies: mouse anti-SMN (BD Biosciences 610647; 1:3,000), anti-HA tag HRP-conjugated (Jackson ImmunoResearch JIM-115-035-003; 1:5000), and anti-ACTB HRP-conjugated (Proteintech HRP-60008-100UL; 1:5,000). Antibodies were diluted in 3% milk in TBS-T (20 mM Tris pH 7.4, 150 mM NaCl, 0.05% Tween-20).
Experiments were performed in three independent biological replicates. Band intensities were quantified using ImageLab (Bio-Rad). Normalization was carried out using the following ratios: HA/control, HA/ACTB, HA/control/ACTB, and SMN/ACTB. Data were plotted in GraphPad Prism and are presented as mean ± SEM of the three independent replicates.

Zebrafish Maintenance

Adult zebrafish and embryos were maintained by standard protocols approved by the University of Queensland and Griffith University Animal Ethics Committee. Ethics approval AE213_18/AE213_18 and GRIDD/11/22/AEC.

hsa-SMN1 VUS c.861_864del (*SMN1-861VUS*) Transgenic Line Generation

5'-mKate-tagged 861VUS cDNA (named *251-pME-mKate2_c.861_864_SMN1* in our database) has been synthesised as gene blocks by Gene Universal *Inc.* and cloned into a Gateway compatible pME-plasmid for subcloning. The *mKate-SMN1-861VUS* sequence was further recombined with the ubiquitin promoter present in pENTR5'_ubi and the previously published destination clone 1455_pDEST_miniTol2_R4-R2_MCS, to generate a final Tol2 DNA transgene for genomic integration, as previously described.[29,31] The plasmid was named *274-UBI-mKate_SMN1-861VUS* in our database. *274-UBI-mKate_SMN1-861VUS* DNA was further injected at 25ng complexed with 25ng of transposase cDNA into one cell stage F0 heterozygous $smn^{Y262stop-/+}$ mutant embryos. mKate/Red positive animals were further selected and grew up to adulthood. One appropriate F0 founder was identified for its ability to transmit the *274-mKate_SMN1-861VUS* transgene into F1 progeny, named $smn^{Y262stop}$;*Tg(UBI-mKate_SMN1-861VUS)*. Homozygous and heterozygous individuals were unknown during the experiments and genotyped by PCR at the end of the experiments. Genomic DNA was amplified by PCR using primers 137-smnY262_FW (GGGTTACATCACCCACCCAA) and reverse primer 156_REVsmnY262-EXT (GACTAGCTAAGCATGCTAACTGGATCAGGCATTACGATAGCAAACGTACAAGAA AAACAACTGTACAAT) in 25 µL reactions, a hybridization temperature of 56 °C. PCR products were verified on a 2% SB agarose gel and subsequently digested with 0.5 µL MluCI at 37 °C overnight. Digested fragments were resolved on a 1% SB agarose gel to determine genotype based on restriction fragment patterns.

Larvae/Juvenile Zebrafish Morphology Analysis

Larvae and juvenile animal morphology was monitored using a MVX10 Macro Zoom Microscope (Olympus, 0.63x) equipped with a DP75 digital camera and controlled with CellSens image analysis software (Olympus). Animals were anesthetized with tricaine and mounted in methylcellulose prior to image acquisition.

Larvae Motor Function Analysis

Larval swimming behavior and response to stress stimuli were assessed using the ZebraBox Revolution system (ViewPoint Life Sciences, France). Larvae were distributed in 24-well plates in triplicate. The behavioral protocol consisted of a 24-minute recording, comprising three cycles of 4 minutes of light and 4 minutes of dark. Data were analyzed using GraphPad Prism (version 9.0.0). Animal position in the 24-well plates was kept the same across the experiments, and genotypes were confirmed by PCR at 25 dpf.
