# Supplementary material for: SMN1 variants identified by false-positive SMA newborn screening tests: Therapeutic hurdles and functional and epidemiological solutions
Source: Am J Hum Genet. 2026 Feb 12;113(3):627–35. doi: 10.1016/j.ajhg.2026.01.012 (PMC13087420; doi:10.1016/j.ajhg.2026.01.012)
Supplement: Document S2. Article plus supplemental information [file mmc3.pdf]

SMN1 variants identified by false-positive SMA newborn screening tests: Therapeutic hurdles and functional and epidemiological solutions

Graphical abstract

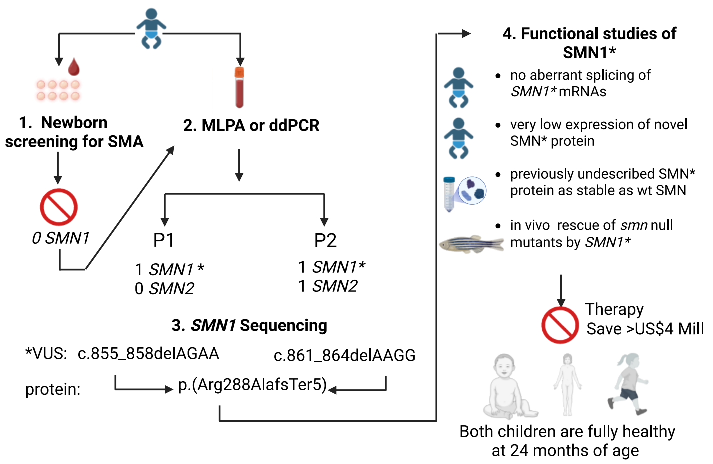

Authors

Brunhilde Wirth, Joyosmita Das, Heike Kölbel, ..., Afsaneh Taghipour-Sheshdeh, Brett W. Stringer, Jean Giacomotto

Correspondence

brunhilde.wirth@uk-koeln.de

Two different 4-base-pair deletions in *SMN1* that cause a similar frameshift led to false-positive SMA newborn screening results. Integrating genomic, functional, and population data clarified diagnosis, preventing unnecessary treatment, and demonstrated that a previously undescribed SMN protein can preserve motor function despite the absence of wild-type full-length SMN.

Wirth et al., 2026, The American Journal of Human Genetics *113*, 627–635
March 5, 2026

© 2026 The Authors. Published by Elsevier Inc. on behalf of American Society of Human Genetics.

https://doi.org/10.1016/j.ajhg.2026.01.012

SMN1 variants identified by false-positive SMA newborn screening tests: Therapeutic hurdles and functional and epidemiological solutions

Brunhilde Wirth,[1,2,3,*] Joyosmita Das,[4] Heike Kölbel,[5] Shuxiang Goh,[6] Michelle A. Farrar,[6,7] Valentina Piano,[1,2] Sebastian Zetzsche,[1,2] Nico Fuhrmann,[1] Jutta Becker,[1] Mert Karakaya,[1,2,3,11] Yougang Zhang,[4] Yuqing Cao,[4] Afsaneh Taghipour-Sheshdeh,[4] Brett W. Stringer,[4] and Jean Giacomotto[4,8,9,10]

Summary

Newborn screening (NBS) for spinal muscular atrophy (SMA) enables rapid diagnosis and pre-symptomatic treatment of infants with bi-allelic *SMN1* deletions. Standard PCR-based assays detect ~95% of cases by identifying the absence of *SMN1* exon 7; however, rare sequence variants can escape detection. We describe two newborns (in Germany and Australia) identified by NBS as lacking *SMN1* but subsequently shown to carry a single *SMN1* copy—with no *SMN2* in P1 and one *SMN2* copy in P2. Gene-specific long-range PCR and Sanger sequencing revealed two distinct 4-bp deletions in *SMN1* exon 7 (c.855_858delAGAA [p.Arg288AlafsTer5] in P1 and c.861_864delAAGG [p.Arg288AlafsTer5] in P2). Both variants disrupt the reverse primer-binding site used in NBS assays and cause the same frameshift p.Arg288AlafsTer5, predicted to be deleterious. A plethora of assays demonstrated preserved exon 7 splicing, markedly reduced SMN protein abundance, and wild-type-like protein thermostability. *In vivo*, expression of the p.Arg288AlafsTer5 protein in zebrafish fully rescued the progressive motor and survival defects of *smn1*-deficient mutants. These findings raise the possibility that this novel SMN isoform has enhanced functional efficiency relative to the wild type. Population data (gnomAD) suggest that ~800 individuals of European ancestry may carry these variants in *trans* with an *SMN1* deletion, yet none have been reported with SMA. Based on our data, no therapy was initiated. Both children remain healthy at 24 months of age, avoiding >US$4 million in potential treatment costs. These findings challenge the assumption that complete loss of full-length SMN invariably causes SMA and suggest that very low levels of this novel SMN isoform can sustain normal motor development.

Spinal muscular atrophy (SMA) is a common autosomal-recessive neuromuscular disorder characterized by progressive degeneration of motor neurons, leading to muscle atrophy. Approximately half of all individuals with bi-allelic *SMN1* (MIM: 600354) loss-of-function (LoF) variants, if left untreated, develop severe SMA type I (MIM: 253300), which is characterized by early death and an inability to sit or walk. The remaining individuals present with intermediate SMA type II (MIM: 253550), in which affected persons are able to sit but not walk, or with milder forms such as SMA type III (MIM: 253400) and, more rarely, adult-onset SMA type IV (MIM: 271150), in which affected persons can sit and walk but often become wheelchair bound as the disease progresses.[1] SMA is caused by bi-allelic loss or mutated survival motor neuron 1 (*SMN1*), while disease severity is largely influenced by *SMN2* (MIM: 601627), a nearly identical copy gene (http://www.omim.org). The *SMN2* copy number varies from 0 to 4 per allele, with higher copy numbers generally conferring milder phenotypes.[2] Most *SMN2* transcripts are alternatively spliced, excluding exon 7, and produce a truncated, unstable protein. Only about 10% of *SMN2* transcripts are full-length, encoding a protein identical to *SMN1*.[3,4]

The recent development of highly effective therapies targeting *SMN1* gene replacement or *SMN2* splicing correction has transformed SMA treatment and management.[5] Newborn screening (NBS) for SMA is now widely implemented,[6] including in Germany[7] and Australia,[8] enabling early diagnosis and prompt initiation of therapy, which significantly improves outcomes.[9,10] The majority of individuals affected by SMA (~95%) are identified by PCR-based assays detecting bi-allelic *SMN1* exon 7 deletions or gene conversions of *SMN1* into *SMN2*.[11–14] However, 3%–5% of individuals with SMA harbor rare sequence variants in *SMN1* that are not detected by standard NBS assays.[2,15,16]

Here, we report two newborns identified by NBS as lacking both *SMN1* copies: one from Germany (individual II-2 from family 1; P1) and one from Australia

[1]Institute of Human Genetics, University of Cologne, Faculty of Medicine, Cologne, Germany; [2]Center for Molecular Medicine Cologne, University of Cologne, Cologne, Germany; [3]Center for Rare Diseases, University Hospital of Cologne, University of Cologne, Cologne, Germany; [4]Institute for Biomedicine and Glycomics, Griffith University, Brisbane, QLD 4111, Australia; [5]Department of Pediatric Neurology, Centre for Neuromuscular Disorders, University Hospital Essen, Essen, Germany; [6]Women's Health, Paediatrics and Child Health, School of Clinical Medicine, University of New South Wales, Sydney, NSW 2033, Australia; [7]Department of Neurology, Sydney Children's Hospital, Randwick, NSW 2031, Australia; [8]School of Environment and Science, Griffith University, Brisbane, QLD 4111, Australia; [9]Thompson Institute, National PTSD Research Centre, University of the Sunshine Coast, Birtinya, QLD 4575, Australia; [10]Queensland Brain Institute, The University of Queensland, Brisbane, QLD 4067, Australia
[11]Present address: Institute of Human Genetics, University Hospital Düsseldorf, Heinrich-Heine-University, Düsseldorf, Germany
*Correspondence: brunhilde.wirth@uk-koeln.de
https://doi.org/10.1016/j.ajhg.2026.01.012.
© 2026 The Authors. Published by Elsevier Inc. on behalf of American Society of Human Genetics.
This is an open access article under the CC BY license (http://creativecommons.org/licenses/by/4.0/).

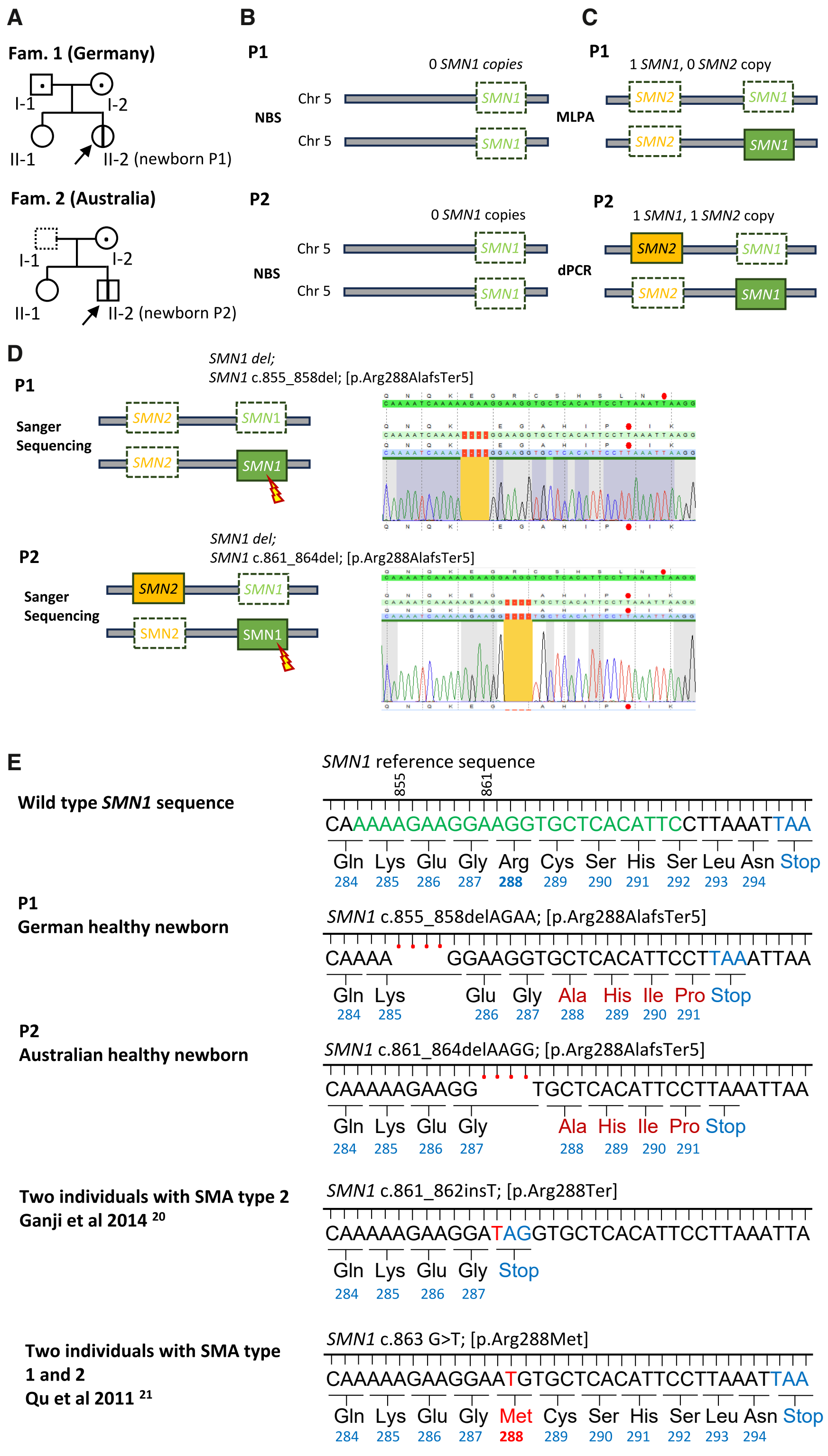

Figure 1. Identification and genetic analysis of two *SMN1*-VUSs in newborns
(A) Pedigrees of the two newborns, P1 and P2. Both were asymptomatic at birth and remained asymptomatic at 2 years of age (indicated by a thick vertical line). The older sisters (II-1) of each newborn were unaffected (open circle). The parents of P1 were carriers (dotted symbols). For P2, the father was unavailable for testing (dashed square), and the mother was a carrier (dotted circle).
(B) Schematic representation of *SMN1* newborn genetic testing using DNA from dried blood spots (DBSs). No *SMN1* signal (blank rectangle) was detected in the *SMN1* exon 7 PCR in either newborn: P1 (from Germany) or P2 (from Australia). For P1, the LightMix TREC SMA HBB kit (Roche) was used; for P2, the Eonis SCID-SMA kit (3234-0010) was employed.
(C) Schematic representation of confirmatory testing using the MRC-Holland P021 MLPA kit (P1) and multiplexing droplet digital PCR (ddPCR) for P2. Both newborns showed one *SMN1* copy (green rectangle). P2 had one *SMN2* copy (orange rectangle), while P1 had none (blank rectangle).
(D) Schematic representation of the SMA alleles in both newborns. A chromatogram of Sanger sequencing shows the *SMN1*-VUS (orange block). Deleted nucleotides are marked by small red rectangles. The entire genomic region spanning the *SMN1* gene was amplified via long-range PCR (exons 1–8)[19] and used as a template to sequence exon 7. A premature stop codon (red dot) is shown in the VUS allele compared to the reference *SMN1* sequence.
(E) Schematic reference *SMN1* exon 7 sequence. NBS primers are indicated in green. Both *SMN1*-VUSs (4-bp deletion, red dots), altered the C-terminal amino acid sequence (red letters), and premature stop codons are shown. Two additional pathogenic variants from individuals with SMA are included for comparison.

(individual II-2 from family 2; P2) (Figures 1A and 1B). Instead, confirmatory testing by multiplex ligation-dependent probe amplification (MLPA; MRC-Holland kit P21-B1[17]) or multiplexing droplet digital PCR (ddPCR)[18] revealed one *SMN1* in both infants, with zero *SMN2* in P1 and one *SMN2* in P2 (Figure 1C). Long-range PCR of genomic *SMN1*[19] followed by Sanger sequencing identified distinct variants of uncertain significance (VUSs) in exon 7 in each newborn: in P1, a 4-bp deletion was detected (*SMN1*: c.855_858delAGAA [GenBank: NM_000344.4] [p.Arg288AlafsTer5], abbreviated 855VUS). In P2, a different 4-bp deletion was identified (*SMN1*: c.861_864delAAGG [GenBank: NM_000344.4] [p.Arg288AlafsTer5], abbreviated 861VUS) (Figure 1D). No further variants were detected in the remaining *SMN1* exons. Both deletions overlap the NBS assay's reverse primer-binding site, explaining the absence of an *SMN1* result (Figure 1E). Haplotype analysis showed that the c.855_858delAGAA variant in P1 was paternally inherited, while the maternal allele carried a deletion. In P2, only the mother was available, and she was a carrier

of the *SMN1* deletion. P2 has one older sister, who does not carry the *SMN1* variant allele; no further relatives were available for this study (Figure 1A).

Both *SMN1* variants result in the same frameshift mutation: p.Arg288AlafsTer5, altering the C-terminal region and introducing a premature stop codon (Figure 1E). These variants were classified by both genetic labs as VUSs. Possible pathogenicity was initially supported by prior reports of pathogenic mutations near the same region in individuals with SMA: two Iranian individuals with SMA type II with a 1-bp insertion (c.861_862insT [p.Arg288Ter])[20] and two Chinese individuals with SMA types I and II carrying a missense variant (c.863G>T [p.Arg288Met]), which caused complete *SMN1* exon 7 skipping (Figure 1D).[21]

The identification of these *SMN1* variants in two clinically healthy newborns presented a major clinical and diagnostic dilemma. Most striking was the observation that P1, who has no *SMN2* copies and hence no known source of wild-type (WT) SMN protein, remained completely asymptomatic. It has long been assumed—supported by mouse models—that a minimum amount of WT, full-length SMN protein is required for survival.[22,23] Therefore, a proband such as P1 with no functional *SMN1* and no *SMN2* copies would result in a miscarriage or stillbirth. P1's asymptomatic state essentially proved that this altered *SMN1* variant has at least some residual function.

Given that the p.Arg288Met mutation was previously shown to severely affect splicing,[21] we investigated the local chromatin regulatory landscape provided by ENCODE. Hi-C data indicate a strong *cis*-regulatory element in this region (Figure 2A). We established lymphoblastoid cell lines from both probands to assess *SMN1* splicing. Sanger sequencing of exon 5–8 cDNA amplicons from P1's RNA revealed a clear sequence of full-length *SMN1*, excluding alternatively spliced transcripts, and provided additional evidence that only *SMN1* is present in this newborn (Figure 2B). In P2, *SMN1* and *SMN2* transcripts were distinguished by DdeI digestion, which cuts specifically in *SMN2* exon 8.[3] *SMN1* transcripts from the variant allele were only full-length, indicating no impact on splicing (Figure 2C).

Western blot analysis of lymphoblastoid cell lysates from P1 and P2, family members of P1, individuals with SMA type IV, SMA carriers, and control subjects showed that both P1 and P2 produced extremely low SMN protein levels—lower than all other samples tested (Figure 3A; supplemental information). Despite this, both children remained clinically well.

Despite the frameshift altering the C-terminal region (residues 282–295), the SMN VUS preserves the glycine and histidine residues that are well conserved in mammals and suggest a potential mammalian-specific function (Figure 3B). To predict the impact of the mutation on SMN 3D structure and stability, we used AlphaFold3[26] (https://alphafoldserver.com) and input the SMN C-terminal region as homo-tetramer or octamer (Figures 3B and S2; supplemental information).[27,28] The α helix oligomerization domain[29] (Figure 3B) is mildly altered by the p.Arg288AlafsTer5 variant but not by p.Arg288Ter (Figure 3C).

We then expressed HA-tagged constructs of WT *SMN1-WT*, or mutated *SMN1*-855VUS, and *SMN1*-861VUS in HeLa cells and assessed thermostability by exposing lysates to increasing temperatures (36°C–51°C). All three proteins showed similar thermostability, comparable to actin, a protein well-known for its thermostability,[30] suggesting that the mutated SMN proteins were structurally stable (Figure 3D; supplemental information).

Using support from the Australian Functional Genomics Network, we rapidly adapted a zebrafish model to evaluate the *in vivo* functionality of *SMN1* variants.[31] In this pilot methodological study, bi-allelic *smn1* LoF zebrafish[32] were injected with a range of control and variant mRNA.[31] Known pathogenic *SMN1* variants failed to rescue the *smn1* LoF phenotype. In contrast, the *SMN1*-855VUS and *SMN1*-861VUS mRNA variants fully reproduced the functional complementation obtained with WT or known benign variant mRNAs. Functional complementation was assessed through normal morphology, swimming behavior, and survival during the first week of life, until the transiently injected mRNA and protein were degraded.[31]

To complement this initial transient mRNA supplementation study, here, we generated a stable transgenic line, *Tg*(*UBI-mKate$_2$-SMN1-861VUS*), ubiquitously expressing *SMN1*-861VUS. The transgene was integrated into the genome of *smn1*$^{\mathrm{Y262stop-/+}}$ zebrafish mutants (Figure 4A; supplemental information; Video S1) using Tol2-mediated transgenesis.[33] As previously shown, all homozygous *smn1*$^{\mathrm{Y262stop-/-}}$ mutants exhibited progressive loss of motor function and died before 6 days post-fertilization (dpf).[31] In contrast, the presence of the transgene encoding the p.Arg288AlafsTer5 protein restored normal development, morphology, and motor function, indistinguishable from WT and heterozygous controls (Figures 4B and 4C), confirming that the encoded protein is functional.

Population-level data from gnomAD v.4.1 (806,504 genomes/exomes) showed that both variants are rare and found exclusively in individuals of European ancestry (N = 589,724). The c.855_858delAGAA variant was observed 13 times and the c.861_864delAAGG variant 47 times, all in a heterozygous state (https://gnomad.broadinstitute.org). No homozygotes were reported. This results in a combined carrier frequency of ~1 in 9,828 individuals.

Given an *SMN1* deletion carrier frequency of ~1 in 35 in Europeans,[34] the expected frequency of compound heterozygotes (*SMN1* deletion: *SMN1*-855VUS or *SMN1*-861VUS) is ~1 in 1.37 million, assuming autosomal-recessive inheritance and random mating ($1/35 \times 1/9{,}828 \times 1/4 = 1/1.37$ million). Based on a current estimated world population of approximately 8.2 billion, with ~13.88% (or ~1.13 billion individuals) of European descent, ~800

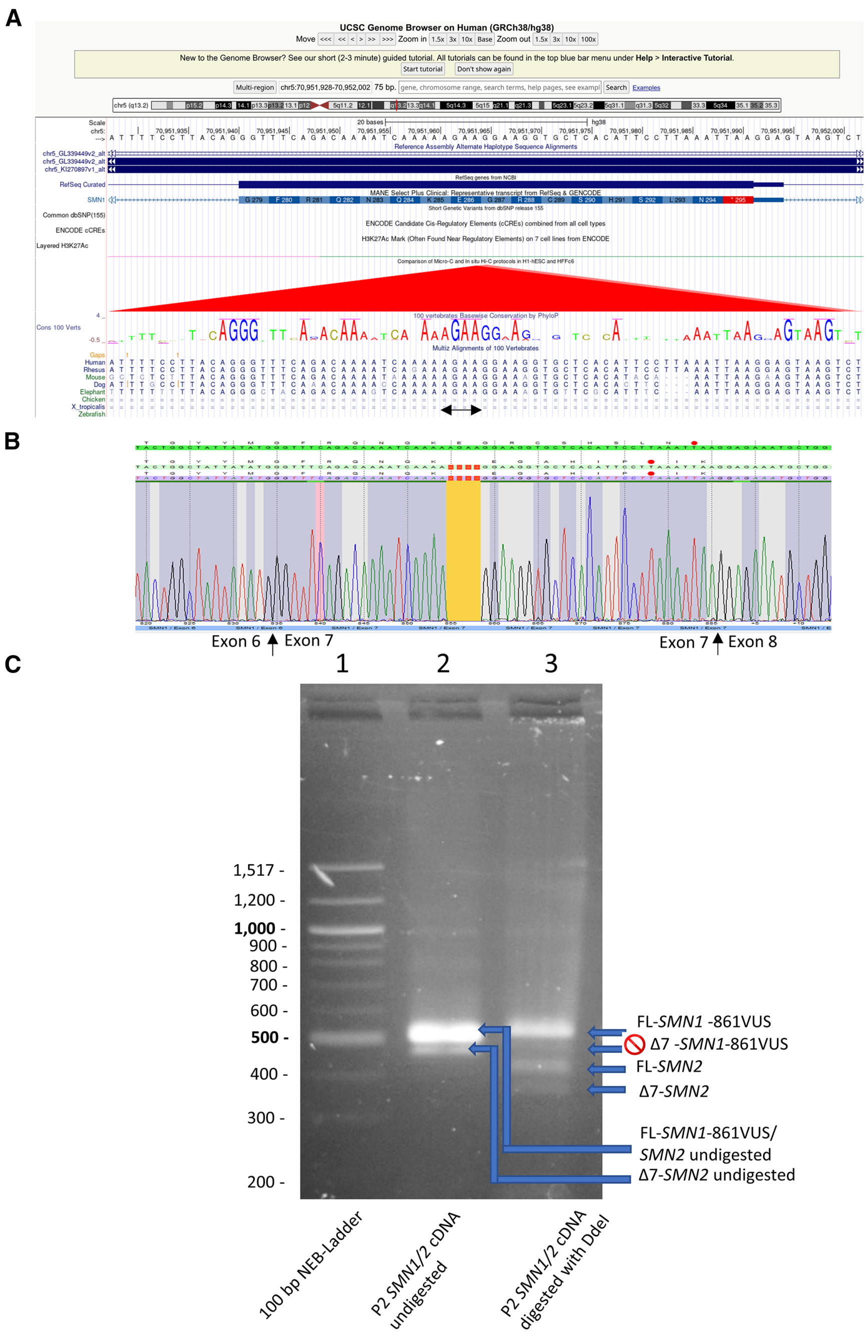

Figure 2. Splicing of *SMN1*-VUS in patient-derived lymphoblastoid cell lines is unaffected

(A) The RNA region chr5:70951940–70951980 (GRCh38/hg38) encompassing the two *SMN1*-VUSs overlaps with a significant Hi-C peak in ENCODE data, suggesting potential regulatory relevance (https://genome.ucsc.edu).

(B) Chromatogram of *SMN1-855VUS* cDNA showing clean exon-exon boundaries between exons 6–7 and 7–8, with no evidence of exon 7 skipping in P1, ruling out aberrant splicing. cDNA was reverse transcribed from total RNA isolated from the Epstein Barr Virus (EBV)-transformed lymphoblastoid cell line of P1 and *SMN1-855VUS* transcripts amplified with primers localized in exons 5 and 8.

(C) PCR analysis of *SMN* transcripts (exons 5–8) after DdeI digestion in P2. For *SMN1-861VUS*, only full-length (FL) transcripts are visible; no Δ7-*SMN1*-861VUS transcripts are detected. In contrast, *SMN2* yields both FL and Δ7 transcripts. cDNA was reverse transcribed from total RNA isolated from the EBV-transformed lymphoblastoid cell line of P2.

(1.13 billion/1.37 million = 824) individuals are predicted to exist who harbor the compound heterozygous genotype—yet none have been reported with SMA, further supporting non-pathogenicity.

The almost simultaneous discovery of the two variants in Germany and Australia aligns with expectations from current NBS coverage. In 2024, ~64% of newborns in the European Union 27 (EU27) were screened for SMA (https://www.sma-europe.eu/newborn-screening-in-sma). In 2023, 3.67 million children were born in the EU27 (https://ec.europa.eu/eurostat/statistics-explained/index.php?title=Fertility_statistics), suggesting that ~2–3 children per year should be compound heterozygous for an *SMN1* deletion with either 855VUS or 861VUS (3.67 million/1.37 million ≈ 2.6).

Additional rare missense variants in primer or probe-binding regions reported in gnomAD v.4.1 (Table S1; Figure S1), ClinVar (Table S2), and NBS programs[11,35–37] may lead to even more false-positive or uncertain NBS results. Considering all 15 reported variants reported in gnomAD within the annealing primer region, of which 9 occur in Europeans, 229 alleles in 227 individuals (112 alleles in 111 Europeans) were identified. This would increase the estimated number of compound heterozygotes in Europeans to ~1,500 (1.13 billion/743,796 ≈ 1,519), with ~5 expected newborns per year in the EU27. Indeed, in both countries, besides the newborns reported here, an additional variant was identified in the initial NBS screening program in Australia,[11] and thus far, the proportion of true-positive results is 96.3%. We are aware of an additional false-positive case in Germany that has not yet been published.

To increase diagnostic accuracy, we recommend that any abnormal result in SMA NBS (bi-allelic absence of *SMN1*) that cannot be confirmed via MLPA or ddPCR should always be followed by complete *SMN1* sequencing, as a known or novel variant within the annealing primer site is the most likely explanation. This combined diagnostic strategy is

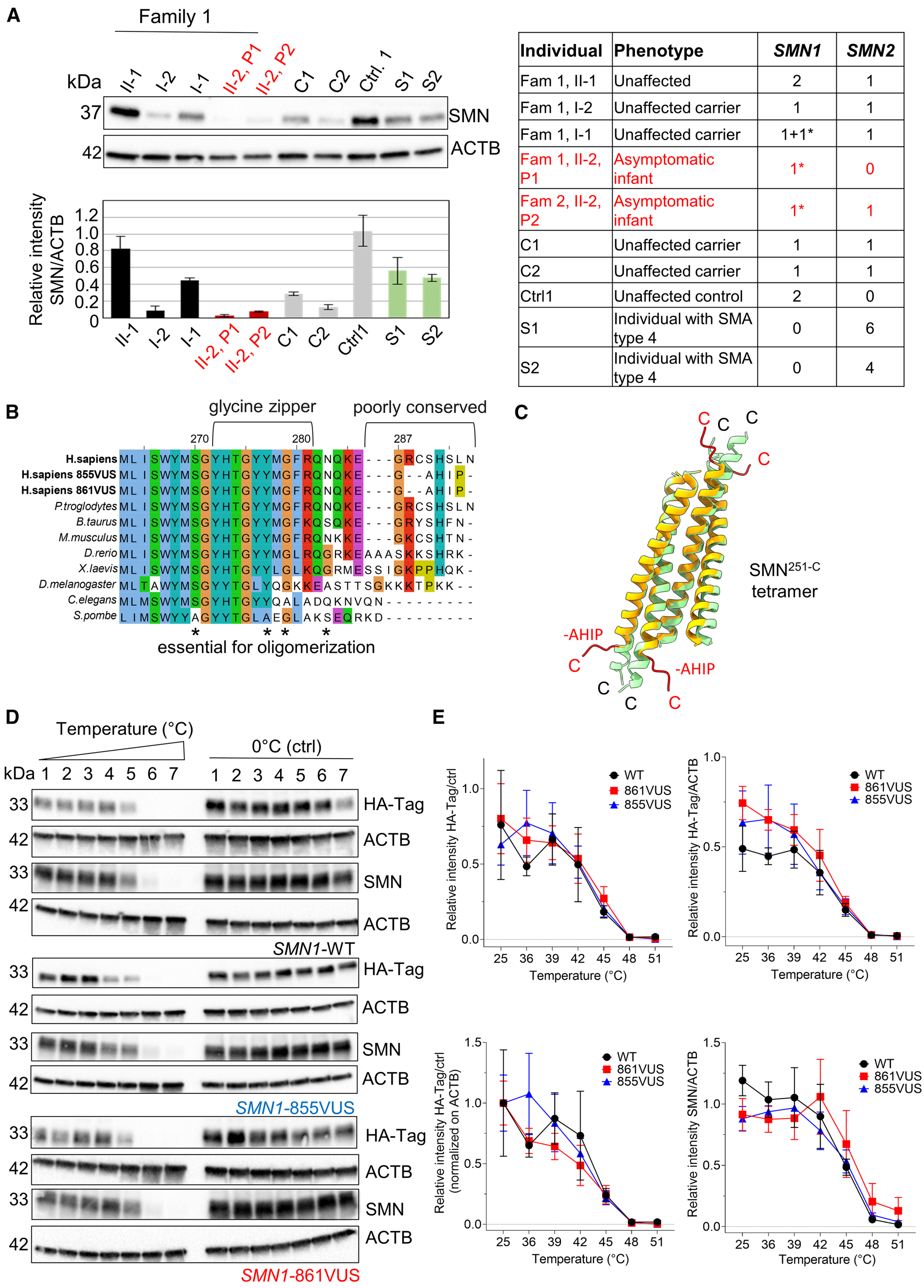

Individual	Phenotype	*SMN1*	*SMN2*
Fam 1, II-1	Unaffected	2	1
Fam 1, I-2	Unaffected carrier	1	1
Fam 1, I-1	Unaffected carrier	1+1*	1
Fam 1, II-2, P1	Asymptomatic infant	1*	0
Fam 2, II-2, P2	Asymptomatic infant	1*	1
C1	Unaffected carrier	1	1
C2	Unaffected carrier	1	1
Ctrl1	Unaffected control	2	0
S1	Individual with SMA type 4	0	6
S2	Individual with SMA type 4	0	4

Figure 3. SMN protein abundance is low in newborns, but SMN-VUS proteins are thermostable

(A) Representative western blot and quantification of protein lysates from EBV-transformed lymphoblastoid cell lines were performed as previously described.[24,25] SMN and actin (ACTB, loading control) levels are shown for P1 and P2, P1 family members, carriers, control, and individuals with SMA type IV. SMN/ACTB ratios are presented as mean ± SD ($n = 4$). Monoclonal antibodies against SMN (BD Biosciences) and ACTB (Proteintech) were used. The corresponding phenotype and *SMN1/SMN2* genotype for each individual are provided in the table on the right.

(B) C-terminal amino acid alignment of SMN across species, highlighting high conservation in the glycine zipper and oligomerization domain (red arrows) and lower conservation in the region containing the SMN-VUS.

(legend continued on next page)

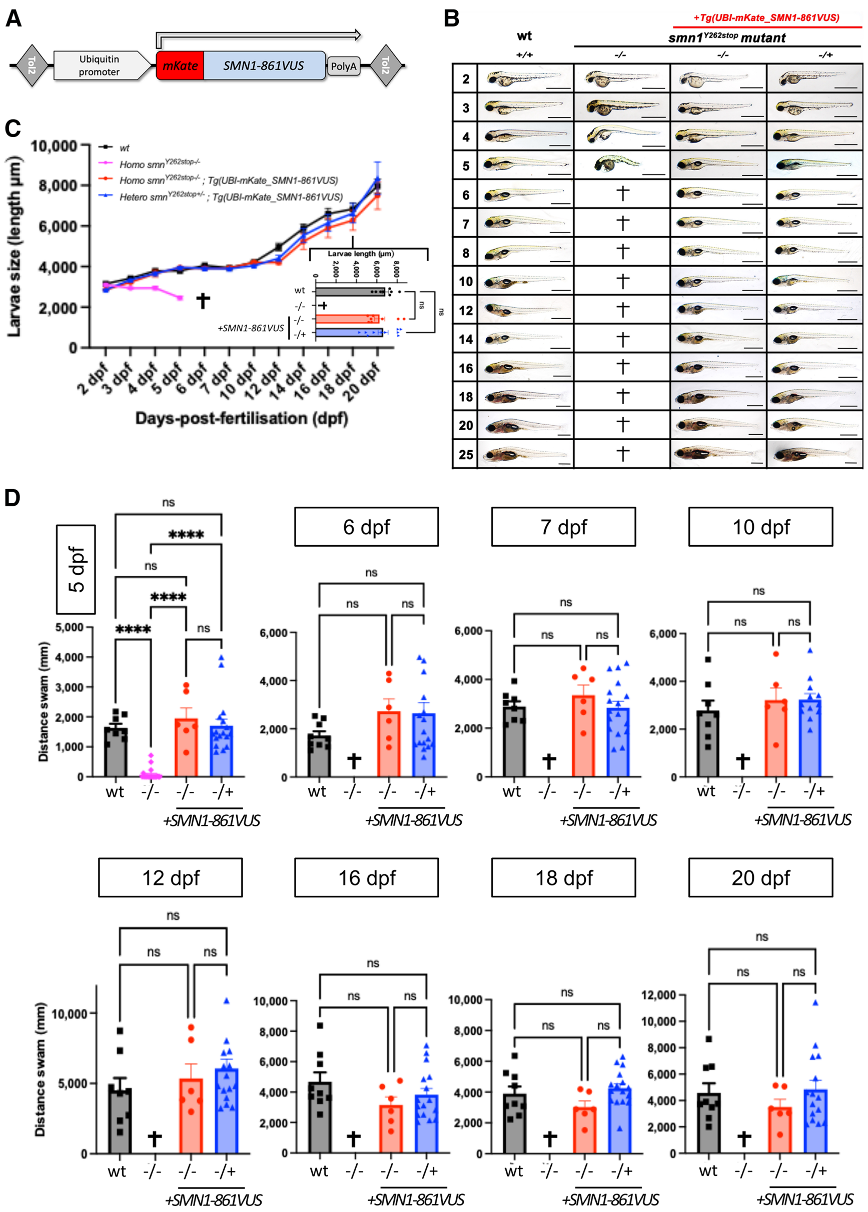

Figure 4. *SMN1-861VUS* transgene fully complements the loss of SMN function in zebrafish

(A) DNA transgene *Tg*(*UBI-mKate_SMN1-861VUS*) integrated into the genome of zebrafish mutants *smn1*$^{Y262stop-/+}$.

(B) Representative images showing morphology from 2 to 25 days post-fertilization (dpf). Homozygous *smn1*$^{Y262stop-/-}$ mutants exhibit progressive deterioration and premature death between 5 and 6 dpf, while expression of *Tg*(*UBI-mKate*$_2$*-SMN1-861VUS*) restores normal development, morphology, and survival. Scale bar: 1,000 μm.

(C) Growth curves showing larval length measurements (±SEM) from 2 to 20 dpf. No significant difference was observed between wild-type (WT), heterozygous, and *Tg*(*UBI-mKate_SMN1-861VUS*)-complemented *smn1*$^{Y262stop-/-}$ animals. The inset (bottom right) highlights comparable body size at 18 dpf.

(D) Motor function comparison from 5 to 20 dpf. Total distance swam (±SEM) during a 24-min recording. Homozygous *smn1*$^{Y262stop-/-}$ mutants display rapid motor function loss culminating in near paralysis by 5 dpf, whereas the transgene *Tg*(*UBI-mKate_SMN1-861VUS*) restores normal locomotor activity indistinguishable from WT and heterozygous controls. Data were analyzed using a non-parametric Kruskal-Wallis test, followed by Dunn's post hoc correction for multiple comparisons; ****$p < 0.0001$.

already implemented in the Australian SMA NBS guidelines (https://doi.org/10.26190/unsworks/31305) and could be adopted globally in such cases.

The strong rescue studies in zebrafish[31] and this study, together with the functional data presented here, as well as the population data, argue that these two VUS alleles are likely non-pathogenic. This assumption is further supported by the analysis of hypomorphic *SMN1* alleles in SMA zebrafish, which—contrary to both *SMN1* VUSs—are unable to fully rescue survival.[31] We speculate that the novel SMN protein retains function and may even possess gain-of-function properties, as it supports survival despite very low abundance.

For P1, gene therapy with Zolgensma was the only potential therapeutic option, as no *SMN2* copies were present. However, since the child already expressed a previously undescribed functional SMN protein and lacked prior exposure to WT SMN, the risk of an immune response was uncertain. For P2, gene therapy with Zolgensma or splice-modifier therapy with nusinersen or risdiplam would have been an option but with limited efficacy expected for the latter, given only one *SMN2* copy.[9,38,39]

(C) AlphaFold3 structural predictions show that the SMN-VUS (orange) has a shorter helix and mildly altered tetramerization compared to SMN wild-type (WT) in green.
(D) Thermostability assay of HA-tagged *SMN1*-WT, HA-*SMN1*-855VUS, and HA-*SMN1*-861VUS expression constructs, transfected in HeLa cells. Protein lysates were prepared after heating from 36°C to 51°C or kept on ice (control). Actin (ACTB), a well-known thermostable protein, was used as a reference. Western blot and immunostaining were performed using HA (Jackson ImmunoResearch), SMN (BD Biosciences), and ACTB (Proteintech) antibodies.
(E) Quantifications of the Western blots included HA/control, HA/ACTB, HA/control/ACTB, and SMN/ACTB. SEM from four independent experiments showed no significant differences between SMN-WT and SMN-VUS proteins.

Ultimately, after reviewing all functional and epidemiological evidence, neither child was started on therapy. Now, at 24 months of age (at the date of submission of the manuscript), both children show normal motor development, confirmed by regular clinical and electrophysiological assessments (supplemental notes). Forgoing treatment avoided patient stress and saved healthcare costs exceeding US $4 million.

In conclusion, our integrated functional, structural, and population-level analyses support a likely non-pathogenic reclassification for the *SMN1* c.855_858delAGAA and c.861_864delAAGG variants. These findings directly influenced clinical decision-making, enabling appropriate care while avoiding unnecessary and costly therapies. Notably, we describe a unique scenario in which very low levels of an altered SMN protein appear to preserve normal motor function despite a complete absence of WT SMN—a condition previously thought incompatible with survival. Detailed functional and cellular studies of this altered protein in the future may uncover enhanced or modified properties and interaction networks, which may be of therapeutic relevance.

Data and code availability

Data generated or analyzed during this study are included in the published article and the corresponding supplemental information. Zebrafish resources used in this study include the previously described *smn1*^{Y262stop} mutant line and a newly generated transgenic line, *Tg*(*UBI-mKate*$_2$*-SMN1-861VUS*). These lines are not deposited in public repositories but are available from Dr. J. Giacomotto upon reasonable request, in accordance with institutional and ethical regulations.

Acknowledgments

We are grateful to both families. This work was funded as follows: Australian Functional Genomics Network Catalyst Grant #11501 to J.G., S.G., and B.W.; a Center for Molecular Medicine Cologne (CMMC) grant to BW (C18); and NHMRC fellowship no. 1174145 to J.G. The Australian Functional Genomics Network is funded by the Medical Research Future Fund (funding ID MRF2007498) and administered by the Murdoch Children's Research Institute.

Author contributions

Conceptualization, B.W. and J.G.; data acquisition, J.D., S.Z., Y.Z., Y.C., A.T.-S., and B.W.S.; data analysis, B.W., J.D., V.P., N.F., J.B., S.Z., H.K., M.A.F., S.G., M.K., and J.G.; funding acquisition, J.G., S.G., and B.W.; visualization, B.W., V.P., and J.G.; clinical data interpretation, H.K., M.A.F., S.G., and M.K.; writing – original draft, B.W.; writing – review and editing, all authors. All co-authors read and approved the final manuscript.

Declaration of interests

The authors declare no competing interests.

Supplemental information

Supplemental information can be found online at https://doi.org/10.1016/j.ajhg.2026.01.012.

Web resources

AlphaFold3, https://alphafoldserver.com
Ensembl, https://www.ensembl.org
Eurostat, https://ec.europa.eu/eurostat/statistics-explained/index.php?title=Fertility_statistics
gnomAD, https://gnomad.broadinstitute.org
OMIM, http://www.omim.org
SMA Europe, https://www.sma-europe.eu/newborn-screening-in-sma
UCSC Genome Browser, https://genome.ucsc.edu

Received: October 18, 2025
Accepted: January 14, 2026
Published: February 12, 2026

References

1. Mercuri, E., Finkel, R.S., Muntoni, F., Wirth, B., Montes, J., Main, M., Mazzone, E., Vitale, M., Snyder, B., Quijano-Roy, S., et al. (2018). Diagnosis and management of spinal muscular atrophy: Part 1: Recommendations for diagnosis, rehabilitation, orthopedic and nutritional care. Neuromuscul. Disord. *28*, 103–115. https://doi.org/10.1016/j.nmd.2017.11.005.
2. Wirth, B., Karakaya, M., Kye, M.J., and Mendoza-Ferreira, N. (2020). Twenty-Five Years of Spinal Muscular Atrophy Research: From Phenotype to Genotype to Therapy, and What Comes Next. Annu. Rev. Genomics Hum. Genet. *21*, 231–261. https://doi.org/10.1146/annurev-genom-102319-103602.
3. Lorson, C.L., Hahnen, E., Androphy, E.J., and Wirth, B. (1999). A single nucleotide in the SMN gene regulates splicing and is responsible for spinal muscular atrophy. Proc. Natl. Acad. Sci. USA *96*, 6307–6311. https://doi.org/10.1073/PNAS.96.11.6307.
4. Wirth, B. (2021). Spinal Muscular Atrophy: In the Challenge Lies a Solution. Trends Neurosci. *44*, 306–322. https://doi.org/10.1016/j.tins.2020.11.009.
5. Mercuri, E., Sumner, C.J., Muntoni, F., Darras, B.T., and Finkel, R.S. (2022). Spinal muscular atrophy. Nat. Rev. Dis. Primers *8*, 52. https://doi.org/10.1038/s41572-022-00380-8.
6. Vrščaj, E., Dangouloff, T., Osredkar, D., Servais, L.; and SMA NBS World Study Group (2024). Newborn screening programs for spinal muscular atrophy worldwide in 2023. J. Neuromuscul. Dis. *11*, 1180–1189. https://doi.org/10.1177/22143602241288095.
7. Vill, K., Schwartz, O., Blaschek, A., Gläser, D., Nennstiel, U., Wirth, B., Burggraf, S., Röschinger, W., Becker, M., Czibere, L., et al. (2021). Newborn screening for spinal muscular atrophy in Germany: clinical results after 2 years. Orphanet J. Rare Dis. *16*, 153. https://doi.org/10.1186/s13023-021-01783-8.
8. Kariyawasam, D.S.T., Russell, J.S., Wiley, V., Alexander, I.E., and Farrar, M.A. (2020). The implementation of newborn screening for spinal muscular atrophy: the Australian

experience. Genet. Med. *22*, 557–565. https://doi.org/10.1038/S41436-019-0673-0.
9. Nishio, H., Niba, E.T.E., Saito, T., Okamoto, K., Takeshima, Y., and Awano, H. (2023). Spinal Muscular Atrophy: The Past, Present, and Future of Diagnosis and Treatment. Int. J. Mol. Sci. *24*, 11939. https://doi.org/10.3390/IJMS241511939.
10. Schwartz, O., Vill, K., Pfaffenlehner, M., Behrens, M., Weiß, C., Johannsen, J., Friese, J., Hahn, A., Ziegler, A., Illsinger, S., et al. (2024). Clinical Effectiveness of Newborn Screening for Spinal Muscular Atrophy: A Nonrandomized Controlled Trial. JAMA Pediatr. *178*, 540–547. https://doi.org/10.1001/jamapediatrics.2024.0492.
11. D'Silva, A.M., Kariyawasam, D.S.T., Best, S., Wiley, V., Farrar, M.A., , et al.; NSW SMA NBS Study Group, Mowat, D., Sampaio, H., Alexander, I.E., Russell, J. (2022). Integrating newborn screening for spinal muscular atrophy into health care systems: an Australian pilot programme. Dev. Med. Child Neurol. *64*, 625–632. https://doi.org/10.1111/dmcn.15117.
12. Czibere, L., Burggraf, S., Fleige, T., Glück, B., Keitel, L.M., Landt, O., Durner, J., Röschinger, W., Hohenfellner, K., Wirth, B., et al. (2020). High-throughput genetic newborn screening for spinal muscular atrophy by rapid nucleic acid extraction from dried blood spots and 384-well qPCR. Eur. J. Hum. Genet. *28*, 23–30. https://doi.org/10.1038/s41431-019-0476-4.
13. Taylor, J.L., Lee, F.K., Yazdanpanah, G.K., Staropoli, J.F., Liu, M., Carulli, J.P., Sun, C., Dobrowolski, S.F., Hannon, W.H., and Vogt, R.F. (2015). Newborn blood spot screening test using multiplexed real-time PCR to simultaneously screen for spinal muscular atrophy and severe combined immunodeficiency. Clin. Chem. *61*, 412–419. https://doi.org/10.1373/clinchem.2014.231019.
14. Kay, D.M., Stevens, C.F., Parker, A., Saavedra-Matiz, C.A., Sack, V., Chung, W.K., Chiriboga, C.A., Engelstad, K., Laureta, E., Farooq, O., et al. (2020). Implementation of population-based newborn screening reveals low incidence of spinal muscular atrophy. Genet. Med. *22*, 1296–1302. https://doi.org/10.1038/S41436-020-0824-3.
15. Costa-Roger, M., Blasco-Pérez, L., Gerin, L., Codina-Solà, M., Leno-Colorado, J., Gómez-García De La Banda, M., Garcia-Uzquiano, R., Saugier-Veber, P., Drunat, S., Quijano-Roy, S., and Tizzano, E.F. (2024). Complex SMN Hybrids Detected in a Cohort of 31 Patients with Spinal Muscular Atrophy. Neurol. Genet. *10*, e200175. https://doi.org/10.1212/NXG.0000000000200175/SUPPL_FILE/ETABLE.
16. Wirth, B. (2000). An update of the mutation spectrum of the survival motor neuron gene (SMN1) in autosomal recessive spinal muscular atrophy (SMA). Hum. Mutat. *15*, 228–237. https://doi.org/10.1002/(SICI)1098-1004(200003)15:3<228::AID-HUMU3>3.0.CO;2-9.
17. Schorling, D.C., Becker, J., Pechmann, A., Langer, T., Wirth, B., and Kirschner, J. (2019). Discrepancy in redetermination of SMN2 copy numbers in children with SMA. Neurology *93*, 267–269. https://doi.org/10.1212/WNL.0000000000007836.
18. Jiang, L., Lin, R., Gallagher, S., Zayac, A., Butchbach, M.E.R., and Hung, P. (2020). Development and validation of a 4-color multiplexing spinal muscular atrophy (SMA) genotyping assay on a novel integrated digital PCR instrument. Sci. Rep. *10*, 19892. https://doi.org/10.1038/s41598-020-76893-7.
19. Kubo, Y., Nishio, H., and Saito, K. (2015). A new method for SMN1 and hybrid SMN gene analysis in spinal muscular atrophy using long-range PCR followed by sequencing. J. Hum. Genet. *60*, 233–239. https://doi.org/10.1038/JHG.2015.16.
20. Ganji, H., Nouri, N., Salehi, M., Aryani, O., Houshmand, M., Basiri, K., Fazel-Najafabadi, E., and Sedghi, M. (2015). Detection of intragenic SMN1 mutations in spinal muscular atrophy patients with a single copy of SMN1. J. Child Neurol. *30*, 558–562. https://doi.org/10.1177/0883073814521297.
21. Qu, Y.J., Du, J., Li, E.Z., Yang, Y.L., Zou, L.P., Bai, J.L., Wang, H., Jin, Y.W., and Song, F. (2011). [Point mutation analysis of SMN1 gene in patients with spinal muscular atrophy]. Zhonghua Yi Xue Yi Chuan Xue Za Zhi *28*, 121–126. https://doi.org/10.3760/CMA.J.ISSN.1003-9406.2011.02.001.
22. Schrank, B., Götz, R., Gunnersen, J.M., Ure, J.M., Toyka, K.V., Smith, A.G., and Sendtner, M. (1997). Inactivation of the survival motor neuron gene, a candidate gene for human spinal muscular atrophy, leads to massive cell death in early mouse embryos. Proc. Natl. Acad. Sci. USA *94*, 9920–9925. https://doi.org/10.1073/PNAS.94.18.9920.
23. Iyer, C.C., Corlett, K.M., Massoni-Laporte, A., Duque, S.I., Madabusi, N., Tisdale, S., McGovern, V.L., Le, T.T., Zaworski, P.G., Arnold, W.D., et al. (2018). Mild SMN missense alleles are only functional in the presence of SMN2 in mammals. Hum. Mol. Genet. *27*, 3404–3416. https://doi.org/10.1093/HMG/DDY251.
24. Helmken, C., Hofmann, Y., Schoenen, F., Oprea, G., Raschke, H., Rudnik-Schöneborn, S., Zerres, K., and Wirth, B. (2003). Evidence for a modifying pathway in SMA discordant families: Reduced SMN level decreases the amount of its interacting partners and Htra2-beta1. Hum. Genet. *114*, 11–21. https://doi.org/10.1007/s00439-003-1025-2.
25. Strathmann, E.A., Hölker, I., Tschernoster, N., Hosseinibarkooie, S., Come, J., Martinat, C., Altmüller, J., and Wirth, B. (2023). Epigenetic regulation of plastin 3 expression by the macrosatellite DXZ4 and the transcriptional regulator CHD4. Am. J. Hum. Genet. *110*, 442–459. https://doi.org/10.1016/j.ajhg.2023.02.004.
26. Abramson, J., Adler, J., Dunger, J., Evans, R., Green, T., Pritzel, A., Ronneberger, O., Willmore, L., Ballard, A.J., Bambrick, J., et al. (2024). Accurate structure prediction of biomolecular interactions with AlphaFold 3. Nature *630*, 493–500. https://doi.org/10.1038/S41586-024-07487-W.
27. Gupta, K., Wen, Y., Ninan, N.S., Raimer, A.C., Sharp, R., Spring, A.M., Sarachan, K.L., Johnson, M.C., Van Duyne, G.D., and Matera, A.G. (2021). Assembly of higher-order SMN oligomers is essential for metazoan viability and requires an exposed structural motif present in the YG zipper dimer. Nucleic Acids Res. *49*, 7644–7664. https://doi.org/10.1093/nar/gkab508.
28. Veepaschit, J., Viswanathan, A., Bordonné, R., Grimm, C., and Fischer, U. (2021). Identification and structural analysis of the Schizosaccharomyces pombe SMN complex. Nucleic Acids Res. *49*, 7207–7223. https://doi.org/10.1093/nar/gkab158.
29. Martin, R., Gupta, K., Ninan, N.S., Perry, K., and Van Duyne, G.D. (2012). The survival motor neuron protein forms soluble glycine zipper oligomers. Structure *20*, 1929–1939. https://doi.org/10.1016/J.STR.2012.08.024.
30. Schüler, H., Lindberg, U., Schutt, C.E., and Karlsson, R. (2000). Thermal unfolding of G-actin monitored with

the DNase I-inhibition assay stabilities of actin isoforms. Eur. J. Biochem. *267*, 476–486. https://doi.org/10.1046/J.1432-1327.2000.01023.X.

31. Stringer, B.W., Yougang, Z., Afsaneh, T.-S., Goh, S., Kölbel, H., Farrar, M.A., Wirth, B., and Giacomotto, J. (2025). Clinical relevance of zebra fish for gene variants testing . Proof-of-principle with SMN1/SMA. EMBO Mol. Med, 1–14. https://doi.org/10.1038/s44321-025-00355-8.
32. Boon, K.L., Xiao, S., McWhorter, M.L., Donn, T., Wolf-Saxon, E., Bohnsack, M.T., Moens, C.B., and Beattie, C.E. (2009). Zebrafish survival motor neuron mutants exhibit presynaptic neuromuscular junction defects. Hum. Mol. Genet. *18*, 3615–3625. https://doi.org/10.1093/hmg/ddp310.
33. Tromp, A., Wang, H., Hall, T.E., Mowry, B., and Giacomotto, J. (2023). Optimising the zebrafish Cre/Lox toolbox. Codon improved iCre, new gateway tools, Cre protein and guidelines. Front. Physiol. *14*, 1221310. https://doi.org/10.3389/FPHYS.2023.1221310/FULL.
34. Feldkötter, M., Schwarzer, V., Wirth, R., Wienker, T.F., and Wirth, B. (2002). Quantitative analyses of SMN1 and SMN2 based on real-time lightcycler PCR: Fast and highly reliable carrier testing and prediction of severity of spinal muscular atrophy. Am. J. Hum. Genet. *70*, 358–368. https://doi.org/10.1086/338627.
35. Akhkiamova, M., Polyakov, A., Marakhonov, A., Voronin, S., Saifullina, E., Vafina, Z., Michalchuk, K., Braslavskaya, S., Chukhrova, A., Ryadninskaya, N., et al. (2024). Rare Variants of the SMN1 Gene Detected during Neonatal Screening. Genes *15*, 956. https://doi.org/10.3390/GENES15070956.
36. Chien, Y.H., Chiang, S.C., Weng, W.C., Lee, N.C., Lin, C.J., Hsieh, W.S., Lee, W.T., Jong, Y.J., Ko, T.M., and Hwu, W.L. (2017). Presymptomatic Diagnosis of Spinal Muscular Atrophy Through Newborn Screening. J. Pediatr. *190*, 124–129.e1. https://doi.org/10.1016/J.JPEDS.2017.06.042.
37. Qu, Y., Bai, J., Jiao, H., Qi, H., Huang, W., OuYang, S., Peng, X., Jin, Y., Wang, H., and Song, F. (2024). Variants located in intron 6 of SMN1 lead to misdiagnosis in genetic detection and screening for SMA. Heliyon *10*, e28015. https://doi.org/10.1016/J.HELIYON.2024.E28015.
38. De Vivo, D.C., Bertini, E., Swoboda, K.J., Hwu, W.L., Crawford, T.O., Finkel, R.S., Kirschner, J., Kuntz, N.L., Parsons, J.A., Ryan, M.M., et al. (2019). Nusinersen initiated in infants during the presymptomatic stage of spinal muscular atrophy: Interim efficacy and safety results from the Phase 2 NURTURE study. Neuromuscul. Disord. *29*, 842–856. https://doi.org/10.1016/J.NMD.2019.09.007.
39. Dangouloff, T., Vrščaj, E., Servais, L., Osredkar, D., , et al.; SMA NBS World Study Group, Aryani, O., Barisic, N., Bashiri, F., Bastaki, L., Benitto, A. (2021). Newborn screening programs for spinal muscular atrophy worldwide: Where we stand and where to go. Neuromuscul. Disord. *31*, 574–582. https://doi.org/10.1016/j.nmd.2021.03.007.

The American Journal of Human Genetics, Volume *113*

Supplemental information

SMN1 variants identified by false-positive SMA newborn screening tests: Therapeutic hurdles and functional and epidemiological solutions

Brunhilde Wirth, Joyosmita Das, Heike Kölbel, Shuxiang Goh, Michelle A. Farrar, Valentina Piano, Sebastian Zetzsche, Nico Fuhrmann, Jutta Becker, Mert Karakaya, Yougang Zhang, Yuqing Cao, Afsaneh Taghipour-Sheshdeh, Brett W. Stringer, and Jean Giacomotto

Supplemental Figure, Tables, Notes and Methods

Figure S1. *SMN1* sequence showing all variants identified in GnomAD in the *SMN1* reverse primer annealing region.

The reverse *SMN1* primer sequence used for NBS testing is depicted in green. Shown are the SNPs and 4-bp deletion variants as well as their frequency in GnomAD. The resulting amino acid exchanges are given in the lower part. The ID accession no: NM_000344.4 (*SMN1*)

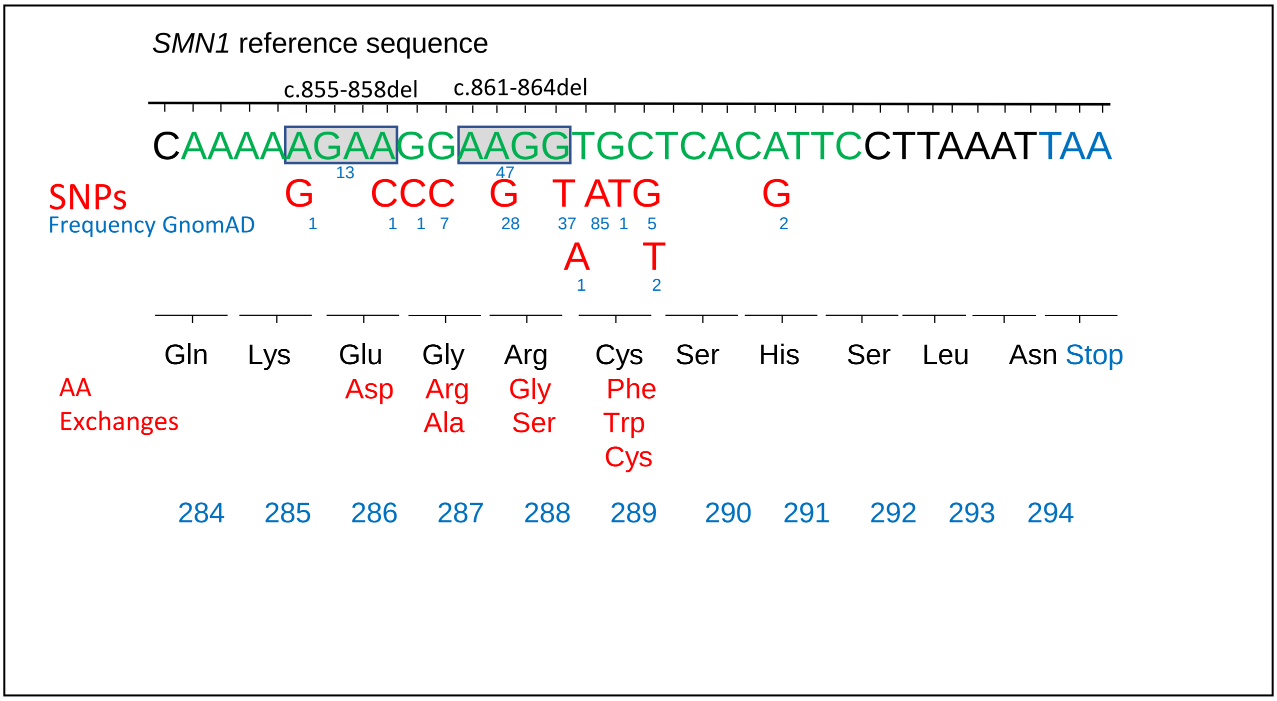

Figure S2. Predicted aligned error (PAE) plots for the SMN WT and SMN1 VUS tetramer and octamer models.

A. PAE plot of the AF3 model of SMN WT tetramer. B. PAE plot of the AF3 model of SMN1 VUS tetramer. C. PAE plot of the AF3 model of SMN WT octamer. D. PAE plot of the AF3 model of SMN1 VUS octamer.

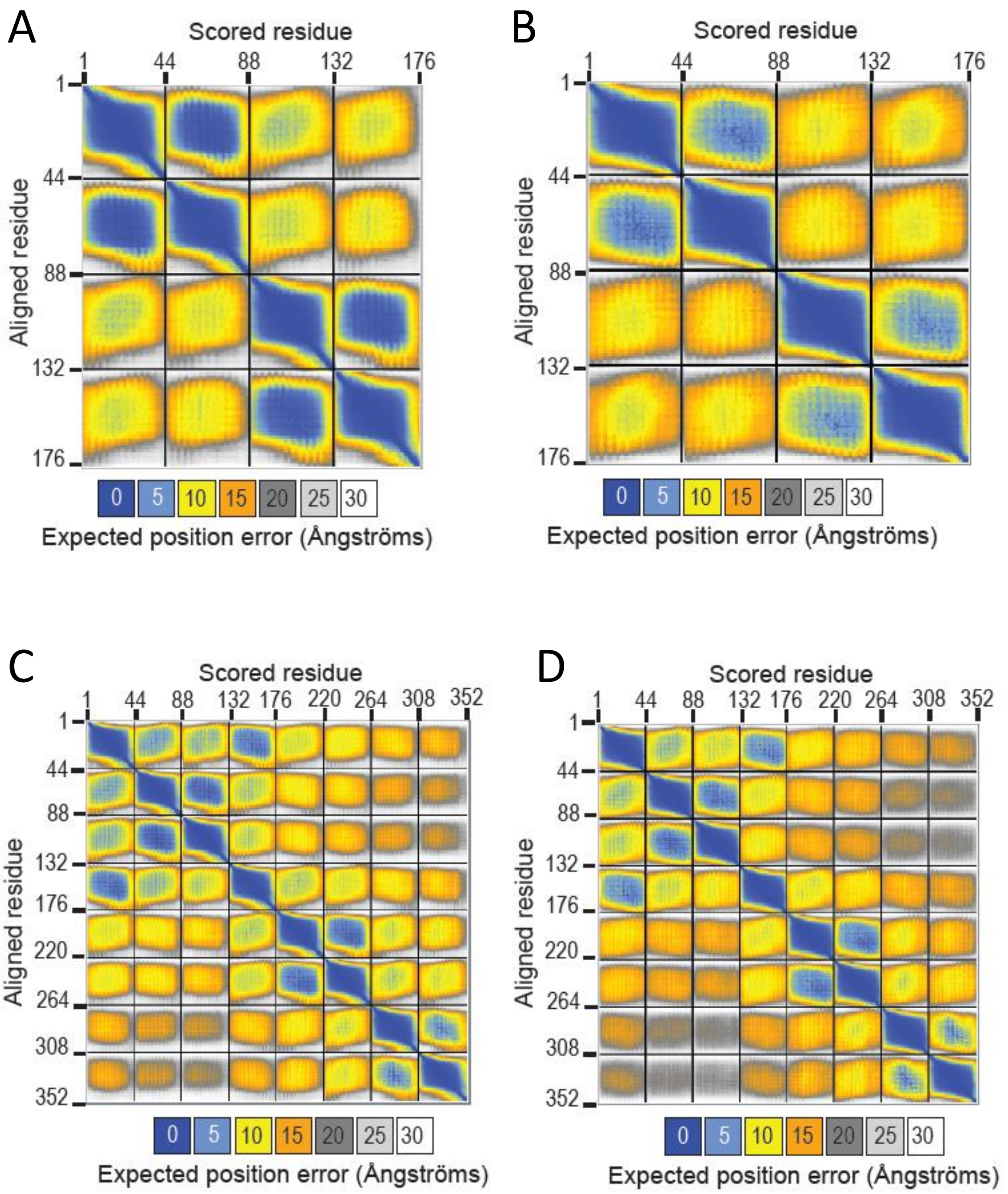

Table S1. Variants reported in gnomAD v4.1 corresponding to the reverse *SMN* primer sequence (chr5:70,951,957-70,951,981) used for NBS testing.

Variant ID = ID in SNP database; Source E= exome, G= genome, HGVS consequence = HGVS protein sequences; VEP=Variant effect predictor; Germline classification= ClinVar germline classification; Allele count is given for all tested E/G and in brackets the counts in the population of European origin. The ID accession no: NM_000344.4 (*SMN1*)

Variant ID	Source	HGVS Consequence	VEP annotation	Germline classification	Allele count (EU)		Allele Number	Allele Frequency	No of homozygous
5-70951957-A-C	E	p.Gln284Pro	missense		3	(3)	1613152	1.86e-6	0
5-70951958-AAAAG-A	G	p.Arg288AlafsTer5	frameshift	Confl. classif. of pathog.	13	(13)	1613162	1.10e-5	0
5-70951961-A-G	E	p.Lys285Lys	synonymous		1	(0)	1613130	6.20e-7	0
5-70951961-AGAAG-A	G	p.Arg288AlafsTer5	frameshift	Confl. classif. of pathog.	47	(47)	1613008	2.91e-5	0
5-70951964-A-C	E	p.Glu286Asp	missense		1	(1)	1613094	6.20e-7	0
5-70951965-G-C	G	p.Gly287Arg	missense	Uncertain significance	1	(0)	1612830	6.20e-7	0
5-70951966-G-C	E	p.Gly287Ala	missense		7	(7)	1613004	4.34e-6	0
5-70951968-A-G	EG	p.Arg288Gly	missense	Uncertain significance	28	(27)	1613040	1.74e-5	1
5-70951970-G-T	EG	p.Arg288Ser	missense	Uncertain significance	37	(0)	1612796	2.29e-5	0
5-70951970-G-A	E	p.Arg288Arg	synonymous		1	(0)	1612918	6.20e-7	0
5-70951971-T-A	EG	p.Cys289Ser	missense	Uncertain significance	85	(9)	1613186	5.27e-5	1
5-70951972-G-T	E	p.Cys289Phe	missense		1	(0)	1613046	6.20e-7	0
5-70951973-C-G	G	p.Cys289Trp	missense		5	(3)	1612820	3.10e-6	0
5-70951973-C-T	G	p.Cys289Cys	synonymous		2	(0)	1612820	1.24e-6	0
5-70951978-A-G	E	p.His291Arg	missense		2	(2)	1613088	1.24e-6	0
**Sum**					229		~1613000	7.04e-4	2
						(112)	1179448	1.05e-5	1

https://genome.ucsc.edu/cgi-bin/hgTracks?db=hg38&lastVirtModeType=default&lastVirtModeExtraState=&virtModeType=default&virtMode=0&nonVirtPosition=&position=chr5%3A70951932%2D70952002&hgsid=2907429012_iAmAMNyWRCnXOiJckcXRmlHPeN0k

Table S2. Variants reported in ClinVar corresponding to the reverse *SMN1* primer sequence (chr5:70,951,957-70,951,981) used for NBS testing.

Link to ClinVar with Variant ID	Molecular consequence	dbSNP ID	no of submitter	clinical significance
VCV000495832 : NM_000344.4(*SMN1*):c.855dup (p.Glu286fs)	frameshift	rs1554082383	1	VUS
VCV001256472 : NM_000344.4(*SMN1):c.855_858del (p.Arg288fs)*	frameshift	rs1475940018	2	VUS
VCV002190592 : NM_000344.4(*SMN1*):c.861_864del (p.Arg288fs)	frameshift	rs1179910122	3	Confl. classif. of path.
VCV003339588 : NM_000344.4(*SMN1*):c.859G>C (p.Gly287Arg)	missense		1	VUS
VCV000644259 : NM_000344.4(*SMN1*):c.862dup (p.Arg288fs)	frameshift	rs1580895068	2	SMA
VCV001949916 : NM_000344.4(*SMN1*):c.862A>G (p.Arg288Gly)	missense	rs1472645065	1	VUS
VCV002988035 : NM_000344.4(*SMN1*):c.862A>T (p.Arg288Trp)	missense	rs1472645065	2	VUS
VCV000928625 : NM_000344.4(*SMN1*):c.864G>T (p.Arg288Ser)	missense	rs368899583	2	VUS
VCV000495833 : NM_000344.4(*SMN1*):c.865T>A (p.Cys289Ser)	missense	rs187925143	2	VUS
VCV000634943 : NM_000344.4(*SMN1*):c.866G>A (p.Cys289Tyr)	missense	rs765273240	1	VUS
VCV003896022 : NM_000344.4(*SMN1*):c.872dup (p.His291fs)	frameshift		1	VUS
VCV000634944 : NM_000344.4(*SMN1*):c.873T>A (p.His291Gln)	missense	rs1561503207	2	VUS

Supplemental Note: Case Reports

P1 (II-2, Family 1) is the second child of non-consanguineous parents from their second pregnancy. The older daughter is healthy and exhibits no developmental concerns. The pregnancy with P1 progressed without complications, with no reported infections and normal fetal movements. Postpartum, following a cesarean section due to breech presentation, she displayed no signs of adjustment disorders, achieving an APGAR score of 10/10. During her initial assessment at 2 weeks of age, she obtained a score of 63 out of 66 on the Children's Hospital of Philadelphia Infant Test of Neuromuscular Disorders (CHOP INTENT), indicating developmentally appropriate performance. By 3 months, she achieved a perfect score of 66 out of 66 in subsequent evaluations. At 9 months, she demonstrated secure alternating crawling, independent sitting, standing with full weight-bearing on her legs, and taking lateral steps. By 12 months, she was able to walk independently. Now, at 24 months of age, she displays normal motor skills, including running, jumping, and climbing. Her language and cognitive development, assessed using the Bayley III scales, also yielded normal results. In the most recent physical examination at 24 months, her muscle tone was normal, with no contractures or signs of muscle atrophy. Deep tendon reflexes were symmetrical, and there were no tongue fasciculations. She demonstrated no difficulties in rising from the ground or climbing stairs. Neurophysiological examinations conducted at all assessments revealed normal latency and amplitude for the median, ulnar, and tibial nerves.

P2 (II-2, Family 2) is the second child of non-consanguineous parents from their second pregnancy. His 3-year-old sister had no concerns for weakness or neurodevelopment. The pregnancy, delivery at term and postpartum periods were unremarkable. Pediatric examination at 2 weeks of age demonstrated good tone and movements, with no proximal weakness. Deep tendon reflexes were present and symmetrical. He was feeding well with a strong suck reflex and no tongue fasciculations. At 3 months, his CHOP-INTEND score was 62/64 and HINE 8. He was able to roll to either side, had upright head control and sat with support. He had good head control on pull to sit with flexed biceps, kicked his legs vigorously and bore weight when held standing. In ventral suspension he extended his neck, back and legs. His Bayley's gross motor assessment scale was 2 standard deviations above the mean.
He continued to make motor progress and at 8 months, he was able to roll on both sides, sit independently and maintain a 4-point crawling position when placed with extended arms. He was starting to shuffle on his tummy short distances in a commando style, bore weight when held standing and bounced up and down easily. He was walking independently at 14 months of age. Longitudinal Bayley's gross motor assessment scale scores and neurophysiology at ages 8, 14, 20 and 26 months demonstrated increasing scores consistent with neurotypical development. In the most recent assessment, he had normal tone, muscle power, and deep tendon reflexes with no tongue fasciculations. He achieved all WHO motor milestones (minus hands and knees crawling), walked independently and was active, with no fatigue, falls or difficulty getting up from the ground. He was speaking in long phrases and short sentences

Supplemental Methods

Ethical standards

Human ethics approval for this work was granted by the Sydney Children's Hospitals Network Human Research Ethics Committee (approval number 2023/ETH01937) and the Ethics Committee of the University of Cologne (approval number 13-022). Informed written consent for the collection of human material, participation in the study and publication purposes was obtained from the legal guardians of the newborns, in accordance with the regulations of the Ethics Committee of the University of Cologne and Sydney.

Western blot analysis of lymphoblastoid cell lines

Epstein–Barr virus (EBV)–transformed lymphoblastoid cell lines were generated from peripheral blood samples by the Wirth laboratory (P1 and family members, further individuals with SMA, SMA carriers, and controls) and the Farrar laboratory (P2) using standard protocols, as previously described.[38,39] Briefly, peripheral blood samples were collected in EDTA as an anticoagulant (1.5 mg EDTA per mL of blood). A volume of 2.5 mL of blood from pediatric donors or 5 mL from adult donors was used. Blood samples were transferred to 50 mL Falcon tubes and brought to a final volume of 50 mL with sterile erythrocyte lysis buffer (155 mM NH_4Cl, 10 mM $KHCO_3$, and 0.1 mM EDTA, pH 7.4). Samples were incubated for 10 min at room temperature and centrifuged at 1,500 rpm for 10 min. This lysis step was repeated twice. Residual red blood cells at the periphery of the white cell pellet were carefully removed using a pipette tip. The remaining cells were resuspended in 2 mL of EBV-containing supernatant and incubated for 1 h at 37 °C. Subsequently, 10 µL cyclosporine A (1 mg/mL; Merck) and 3 mL of RPMI 1640 medium (Gibco) were added, and the cell suspension was transferred to a T25 culture flask. Cultures were maintained at 37 °C, and successful transformation was monitored daily.

EBV-transformed cell lines were maintained in RPMI 1640 supplemented with 20% fetal bovine serum (FBS), penicillin/streptomycin, and amphotericin B, at 37 °C in 5% CO_2. Medium was replenished every 3–4 days based on color change of the pH indicator (from light red to yellow). The day before harvesting for Western blot analysis, fresh medium was added to allow exponential growth of all cell lines.

Western blot analysis was performed as previously described.[38] Cells were harvested in RIPA buffer (150 mM NaCl, 1% NP-40, 0.5% sodium deoxycholate, 0.1% SDS, 50 mM Tris-HCl, pH 8.0) to prepare total protein extracts. Protein concentration was quantified using a Qubit 3 Fluorometer (Thermo Fisher Scientific) and the Qubit Protein Assay Kit (Q33211, Thermo Fisher Scientific). Denatured protein samples (20 µg per sample) were resolved by 10% SDS–PAGE and transferred onto nitrocellulose membranes using the FastBlot Transfer Kit (Bio-Rad). Protein transfer was assessed by Ponceau S staining (Thermo Fisher Scientific).

Membranes were immunostained and signal detection was performed using a chemiluminescence reagent (SuperSignal™ West Pico PLUS, Pierce, Rockford, USA) according to standard protocols. The following primary antibodies were used: mouse monoclonal anti-actin HRP-conjugated antibody (Proteintech HRP-60008-100UL; 1:5,000) and mouse monoclonal anti-SMN antibody (BD Biosciences 610647;

1:2,000). Antibodies were diluted in 3% non-fat dry milk in TBS-T (20 mM Tris-HCl pH 7.4, 150 mM NaCl, 0.05% Tween-20). Each experiment was repeated at least four times, and band intensities were quantified using Fiji software.

AlphaFold 3 predictions

Protein structure predictions were generated using AlphaFold3 (AF3), employing the standard multimodal pipeline integrating sequence, evolutionary, and structural features. Input protein sequences (SMN1 WT and 855/861VUS) either in 4 or 8 copies were submitted to the AF3 prediction server (AlphaFold Server). The top-ranked model was selected for representation using Chimera X software, and confidence metrics (predicted aligned error, PAE) were used to assess local and global reliability of the predictions (**Figure S2**).

Thermostability assay

HeLa cells were maintained at 37 °C in a humidified atmosphere containing 5% CO_2 and cultured in DMEM supplemented with 10% (v/v) fetal bovine serum (FBS) and 0.1× penicillin–streptomycin. Cells (1 × 10^5) were seeded into one well of a 6-well plate. After 18 h, the medium was replaced with DMEM/F-12, and cells were transfected with 0.1 µg/mL plasmid DNA. After 48 h, cells were harvested and resuspended in PBS (10 mM Na_2HPO_4, 1.8 mM KH_2PO_4, 137 mM NaCl, 2.7 mM KCl, pH 7.2).
cDNAs of *SMN1* WT, *SMN1* 855VUS and *SMN1* 861VUS were introduced using Gibson cloning assembly in the backbone plasmid AAV Ef1a (Addgene 135428), fusing the HA tag at the N-terminus of SMN1 via PCR (primer_1_F ATACGATGTTCCAGATTACGCTATGGCGATGAGCAGCG, primer_2_F TCGTGAGGTACCATGTACCCATACGATGTTCCAGA)
For each condition, 50 µL of the cell suspension was aliquoted into PCR tubes. Tubes were either kept on ice (0 °C control) or placed in a thermocycler set to 25, 45, 48, 51, 54, 57, or 60 °C. Samples were incubated for 6 min, followed by 6 min at 4 °C. Cells were then centrifuged and lysed in RIPA buffer supplemented with protease inhibitor cocktail (MilliporeSigma). Lysates were incubated on ice for 30 min and centrifuged at 12,000 rcf for 20 min at 4 °C.
A total of 15 µg protein per sample was loaded for SDS–PAGE and Western blotting. Membranes were probed with the following primary antibodies: mouse anti-SMN (BD Biosciences 610647; 1:3,000), anti-HA tag HRP-conjugated (Jackson ImmunoResearch JIM-115-035-003; 1:5000), and anti-ACTB HRP-conjugated (Proteintech HRP-60008-100UL; 1:5,000). Antibodies were diluted in 3% milk in TBS-T (20 mM Tris pH 7.4, 150 mM NaCl, 0.05% Tween-20).
Experiments were performed in three independent biological replicates. Band intensities were quantified using ImageLab (Bio-Rad). Normalization was carried out using the following ratios: HA/control, HA/ACTB, HA/control/ACTB, and SMN/ACTB. Data were plotted in GraphPad Prism and are presented as mean ± SEM of the three independent replicates.

Zebrafish Maintenance

Adult zebrafish and embryos were maintained by standard protocols approved by the University of Queensland and Griffith University Animal Ethics Committee. Ethics approval AE213_18/AE213_18 and GRIDD/11/22/AEC.

hsa-SMN1 VUS c.861_864del (*SMN1-861VUS*) Transgenic Line Generation

5'-mKate-tagged 861VUS cDNA (named *251-pME-mKate2_c.861_864_SMN1* in our database) has been synthesised as gene blocks by Gene Universal *Inc.* and cloned into a Gateway compatible pME-plasmid for subcloning. The *mKate-SMN1-861VUS* sequence was further recombined with the ubiquitin promoter present in pENTR5'_ubi and the previously published destination clone 1455_pDEST_miniTol2_R4-R2_MCS, to generate a final Tol2 DNA transgene for genomic integration, as previously described.[29,31] The plasmid was named *274-UBI-mKate_SMN1-861VUS* in our database. *274-UBI-mKate_SMN1-861VUS* DNA was further injected at 25ng complexed with 25ng of transposase cDNA into one cell stage F0 heterozygous *smn*$^{Y262stop-/+}$ mutant embryos. mKate/Red positive animals were further selected and grew up to adulthood. One appropriate F0 founder was identified for its ability to transmit the *274-mKate_SMN1-861VUS* transgene into F1 progeny, named *smn*^{Y262stop};*Tg(UBI-mKate_SMN1-861VUS)*. Homozygous and heterozygous individuals were unknown during the experiments and genotyped by PCR at the end of the experiments. Genomic DNA was amplified by PCR using primers 137-smnY262_FW (GGGTTACATCACCCACCCAA) and reverse primer 156_REVsmnY262-EXT (GACTAGCTAAGCATGCTAACTGGATCAGGCATTACGATAGCAAACGTACAAGAA AAACAACTGTACAAT) in 25 µL reactions, a hybridization temperature of 56 °C. PCR products were verified on a 2% SB agarose gel and subsequently digested with 0.5 µL MluCI at 37 °C overnight. Digested fragments were resolved on a 1% SB agarose gel to determine genotype based on restriction fragment patterns.

Larvae/Juvenile Zebrafish Morphology Analysis

Larvae and juvenile animal morphology was monitored using a MVX10 Macro Zoom Microscope (Olympus, 0.63x) equipped with a DP75 digital camera and controlled with CellSens image analysis software (Olympus). Animals were anesthetized with tricaine and mounted in methylcellulose prior to image acquisition.

Larvae Motor Function Analysis

Larval swimming behavior and response to stress stimuli were assessed using the ZebraBox Revolution system (ViewPoint Life Sciences, France). Larvae were distributed in 24-well plates in triplicate. The behavioral protocol consisted of a 24-minute recording, comprising three cycles of 4 minutes of light and 4 minutes of dark. Data were analyzed using GraphPad Prism (version 9.0.0). Animal position in the 24-well plates was kept the same across the experiments, and genotypes were confirmed by PCR at 25 dpf.
